# First synthesis of cryptands with sucrose scaffold

**DOI:** 10.3762/bjoc.15.20

**Published:** 2019-01-23

**Authors:** Patrycja Sokołowska, Michał Kowalski, Sławomir Jarosz

**Affiliations:** 1 Institute of Organic Chemistry, Polish Academy of Sciences, Kasprzaka 44/52, 01-224 Warsaw, Poland

**Keywords:** cryptands, macrocyclization, sucrose

## Abstract

Cryptands with sucrose scaffold, an unknown class of such derivatives, were prepared from the readily available 2,3,3’,4,4’-penta-*O*-benzylsucrose and 1’,2,3,3’,4,4’-hexa-*O*-benzylsucrose.

## Introduction

The design and synthesis of macrocyclic receptors is one of the main challenges of supramolecular chemistry [1–2]. These artificial systems exhibit interesting properties and find many applications in, e.g., selective complexation of ionic and neutral species, medicinal chemistry (as drug carriers), and in the synthesis (as phase transfer catalysts). The complexing properties depend on several crucial parameters such as cavity size, type of heteroatoms involved in the ring, functional groups etc.

Carbohydrates are especially useful platforms for macrocycles being able to recognize enantiomers. Special attention is directed to native and modified cyclodextrins, cyclic oligosaccharides, which have found wide application in many aspects of chemistry and industry [3–4]. There are also reports on the preparation of ‘distorted’ cyclodextrins in which diverse fragments are incorporated into the original oligosaccharide ring(s) [5].

Another class of sugar receptors is represented by macrocyclic derivatives with the carbohydrate unit being a part of a crown or aza-crown structure. Up to date, only monosaccharides have been intensively used as chiral building blocks in the synthesis of such macrocycles. Less attention, however, has been paid to macrocycles with disaccharides (or oligosaccharides) being a part of the ring [6–8].

**Figure 1:**
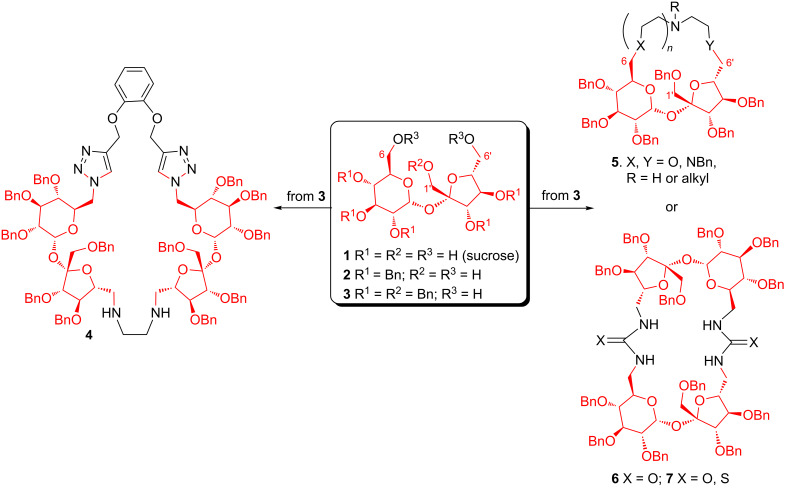
Macrocyclic derivatives with sucrose scaffold.

We have proposed to use sucrose (**1**) as a convenient chiral platform for such macrocycles [9–10]. Several of them, such as **5** (Figure 1), are able to differentiate enantiomers of α-phenylethylammonium salt [11–12]. Sucrose dimers containing two urea or thiourea units (**6** or **7**) are able to complex anions [13–14]. More complex derivatives with sucrose scaffold (e.g., **4**) can be also prepared successfully [15–16].

All macrocyclic derivatives, shown in Figure 1, were prepared from hexa-*O*-benzylsucrose **3** by a connection of the terminal positions of glucose (C6) and fructose (C6’) units.

## Results and Discussion

**Figure 2:**
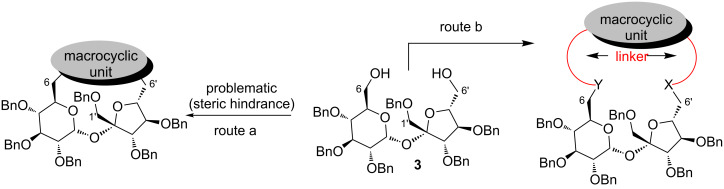
Strategy to sucrose cryptands with additional macrocyclic unit.

Diol **3** may be also used as a starting material for the preparation of cryptands containing a sucrose platform, a completely new class of such derivatives. This goal can be achieved by introduction of an additional macrocyclic unit connecting both terminal positions as shown in Figure 2. The direct connection of both CH_2_–OH groups (route a in Figure 2) is, however, problematic, because of steric hindrance.

The approach shown on route b (Figure 2), in which the terminal positions are elongated to avoid these difficulties, seems to be more feasible.

**Scheme 1:**
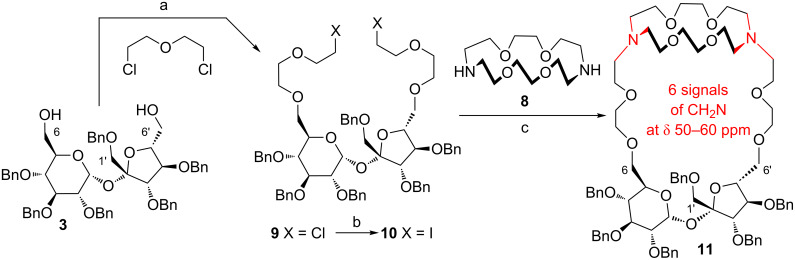
a) 50% NaOH, Bu_4_NHSO_4_, 74%; b) NaI, acetone, 95%; c) Na_2_CO_3_, ACN, 80 °C, 24 h, 33%.

Diol **3** was, thus, extended at both terminal positions by five atoms by reaction with bis(2-chloroethyl) ether; this process provided an intermediate **9** in good yield. Replacement of both terminal chlorine atoms by iodine afforded compound **10**, which was reacted with commercially available diaza-crown ether **8** to afford the first sucrose cryptand **11** in excellent yield (33%, Scheme 1).

The structure of this first sucrose cryptand, suggested by HRMS analysis [*m*/*z =* 1285.6803 which corresponds to (C_74_H_97_N_2_O_17_ + H^+^)], was confirmed by the NMR data. In the ^13^C NMR spectrum of the final compound, six characteristic signals at δ ≈ 50–60 ppm were observed. They can be assigned to the methylene groups connected to the nitrogen atoms (-CH_2_N units), and thus this observation confirms the presence of the crown-unit **8** in the structure.

Although the first approach to sucrose cryptands was very successful, the strategy based on sucrose diol **3** has several limitations. First of all, the cavity in cryptand **11** is large and – since it is accessible from both sides of the molecule – it may decrease the complexing properties especially of chiral guests.

**Figure 3:**
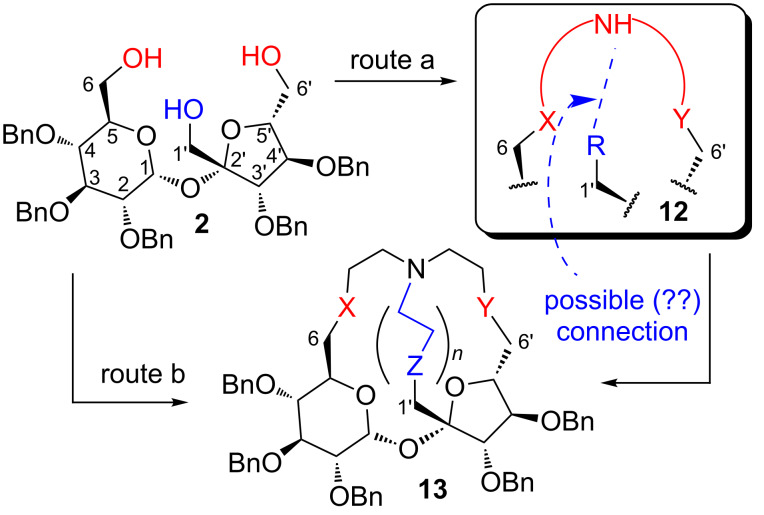
A concept for synthesis of a cryptand from penta-*O*-benzylsucrose (**2**).

To differentiate substantially both sides of the molecule, another type of cryptand is needed, in which all three terminal positions (C1’, C6, C6’) are connected. This goal can be realized starting from sucrose triol **2** as shown in Figure 3.

The first approach consists of a connection of the C1’-position of an intermediate aza-crown ether **12** with the secondary nitrogen atom present in a linker (route a in Figure 3). Another one (route b in Figure 3) involves a direct coupling of all three positions: C1’,C6, C6’.

Recently we have proposed a convenient method for the conversion of triol **2** into a number of sucrose macrocycles having various substituents at the C1’-position [17–18]. Modification of these synthetic routes should allow, eventually, the preparation of derivative **12** in which the C1’ and the ring nitrogen atom might be connected to form cryptand **13** (route a; Figure 3). However, we have found that the preparation of a suitable aza-crown intermediate **12** with the secondary amine function in the linker and the properly modified C1’-position caused substantial problems. We decided, therefore, to prepare sucrose cryptands according to route b, i.e., to connect all three terminal positions at the same time.

**Scheme 2:**
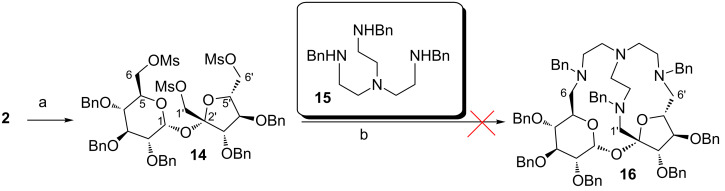
a) MsCl, Et_3_N, DMAP, DCM, −78 °C to rt.; b) Na_2_CO_3_, KI, ACN, reflux.

The first attempt was, however, unsuccessful. Activation of all terminal hydroxy groups as mesylates and subsequent reaction of such intermediate **14** with tripodal amine **15** [19] did not provide the desired cryptand **16** (Scheme 2); the starting material remained unchanged.

**Scheme 3:**
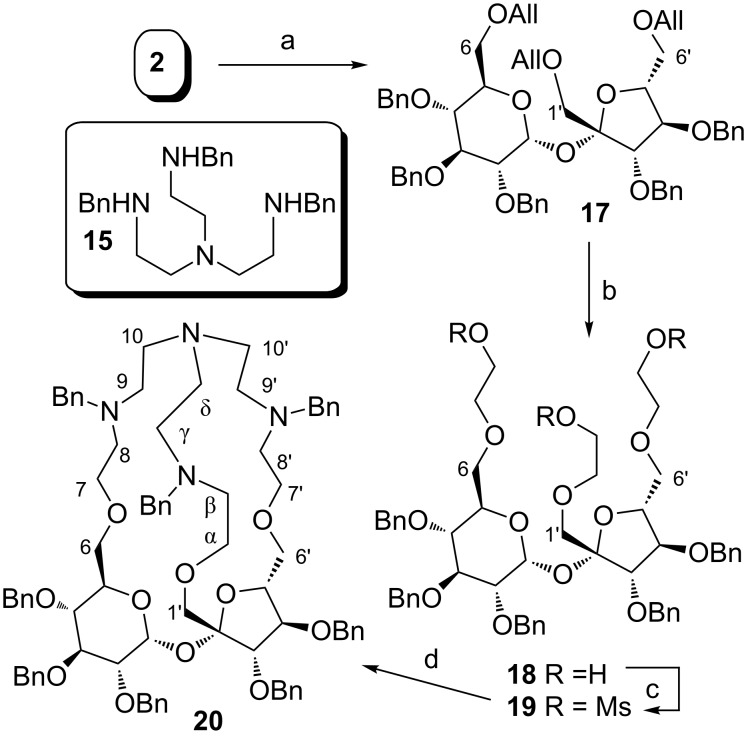
a) AllBr, TBAB, PhMe, 50% NaOH, 50 °C, 18 h, 94%; b) i. O_3_, DCM, −78 °C; ii. NaBH_4_, DCM, MeOH, rt, 16 h; c) MsCl, Et_3_N, DMAP, DCM, −78 °C to rt, 43% over 3 steps; d) Na_2_CO_3_, KI, ACN, **15**, reflux.

This fact may result either from the steric hindrance or the very low reactivity of the hydroxymethylene group [20–21]. We decided, therefore, to elongate the sucrose skeleton by two carbon atoms at the C-1’ position as well as at other terminal positions. Thus, all three hydroxy groups were protected as allyl ethers which were subsequently converted into -CH_2_CH_2_OH units (Scheme 3). Activation of the free OH groups in **18** as mesylates **19** and subsequent reaction with tripodal amine **15** afforded cryptand **20** (Scheme 3). Its presence was confirmed by HRMS [*m*/*z =* 1287.7020 which corresponds to (C_80_H_94_N_4_O_11_ + H^+^)] but the NMR spectrum of **20** was difficult for interpretation because it showed very dynamic changes. Many signals in the ^1^H NMR as well as ^13^C NMR spectra overlapped which made the full analysis difficult. However, the presence of three characteristic signals at δ ≈ 60 ppm in the ^13^C NMR spectrum, which can be assigned to the -N*CH_2_*Ph fragments, fully confirmed the presence of a tripodal amine **15** fragment in the structure of the cryptand.

The method presented in Scheme 3 can be also applied to prepare cryptands with larger cavities. This required an elongation of all three terminal positions (at C1’,C6,C6’) by a longer linker. Thus, reaction of triol **2** with bis(chloroethyl) ether provided derivative **21** in good yield. Replacement of the chlorine atoms for iodine gave **22** which was subjected to the reaction with tripodal amine **15**.

**Scheme 4:**
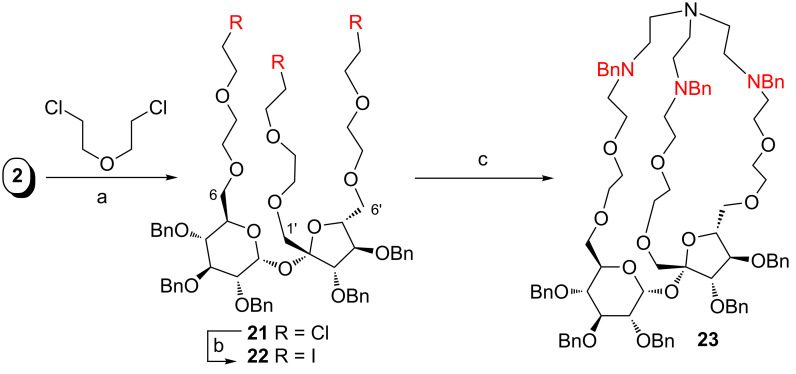
a) 50% NaOH, Bu_4_NHSO_4_, 58%; b) NaI, acetone, 95%; c) Na_2_CO_3_, ACN, 80 °C, 24 h, 45.5%.

The cyclization reaction provided cryptand **23** in very high yield: 45.5% (Scheme 4). The structure of the cryptand was confirmed by HRMS [*m/z* = 1419.7803 which corresponds to (C_86_H_107_N_4_O_14_ + Na^+^)]. Further confirmation came from the ^13^C NMR data. In the spectrum recorded in acetone-*d*_6_, three signals at δ ≈ 60 ppm were seen. These signals were assigned (by HSQC experiments) to the benzyl groups connected to nitrogen atoms (-N*C*H_2_Ph), thus finally confirming the presence of a tripodal unit in the structure of the cryptand.

## Conclusion

We proposed convenient routes to a completely new class of macrocyclic derivatives: cryptands with sucrose scaffold. Such cryptands can be prepared either from a ‘sucrose diol’ (hexa-*O*-benzylsucrose) by introduction of an additional macrocyclic unit connecting the terminal positions, or ‘sucrose triol’ (penta-*O*-benzyl-sucrose). The latter approach is probably more convenient since it allows preparing cryptands of various size of cavity just by simple elongation of all three terminal positions with linkers of different size. In our model studies we have elongated these positions with the same linker. However, since all three terminal positions can be differentiated (as we have proven during our earlier studies see ref. [10]), it is possible to introduce different linkers at the C1’, C6, and C6’ fragments. Thus, our first syntheses of cryptands from ‘sucrose triol’ may open a possibility to prepare such structures with different size depending on the need.

## Experimental

### General

NMR spectra were recorded in CDCl_3_ (internal Me_4_Si) with a Varian AM-600 (600 MHz ^1^H, 150 MHz ^13^C) spectrometer at rt unless otherwise stated. Chemical shifts (δ) are reported in ppm relative to Me_4_Si (δ 0.00) for ^1^H and residual chloroform (δ 77.00) for ^13^C. All significant resonances (carbon skeleton) were assigned by COSY (^1^H–^1^H), HSQC (^1^H–^13^C), and HMBC (^1^H–^13^C) correlations. Reagents were purchased from Sigma-Aldrich, Alfa Aesar or ABCR, and used without purification. Hexanes (65–80 °C fraction from petroleum) and EtOAc were purified by distillation. All other commercially available solvents were used without purification. Thin-layer chromatography was carried out on silica gel 60 F_254_ (Merck). Column chromatography was performed on silica gel 60 (70–230 mesh, Merck). Flash chromatography was performed on Büchi glass columns packed with silica gel 60 (230–400 mesh, Merck), using a Knauer Smartline system with a Büchi fraction collector. The organic solutions were dried over MgSO_4_ or Na_2_SO_4_. Optical rotations were measured with a Jasco P 1020 polarimeter (sodium light) in chloroform at room temperature.

**1’,2,3,3’,4,4’-Hexa-*O*-benzyl-6,6’-bis[2-(2-chloroethoxy)ethyl]sucrose (9):** A solution of compound **3** (100 mg, 0.11 mmol) and tetrabutylammonium hydrogen sulfate (38.5 g, 0.11 mmol) in bis(2-chloroethyl) ether (284 µL, 2.42 mmol) was vigorously stirred with 50% NaOH solution (423 µL) at room temperature for 3 h. Then CH_2_Cl_2_ (1.5 mL) and water (1.5 mL) were added, the organic layer was separated and the aqueous one extracted with CH_2_Cl_2_ (2 × 5 mL). The combined organic solutions were washed with water (2 × 5 mL), dried, and concentrated under high vacuum to remove excess of bis(2-chloroethyl) ether. The crude material was purified by column chromatography (hexane/ethyl acetate 80:20) to afford the title product **9** (92 mg, 0.08 mmol, 74%) as an oil. [α] = +29.9; ^1^H NMR δ 5.66 (d, *J*_1,2_ = 3.6 Hz, 1H, H-1), 4.90 (d, *J* = 10.9 Hz, 1H, OC*H*_2_Ph), 4.86 (d, *J* = 11.0 Hz, 1H, OC*H*_2_Ph), 4.76 (d, *J* = 10.9 Hz, 1H, OC*H*_2_Ph), 4.67 (d, *J* = 11.4 Hz, 1H, OC*H*_2_Ph), 4,64 (dd, 2H, OC*H*_2_Ph), 4.58–4.54 (m, 4H, OC*H*_2_Ph), 4.52 (d, *J* = 11.4 Hz, 1H, OC*H*_2_Ph), 4.44–4.40 (m, OC*H*_2_Ph, 2H, H-3’), 4.10 (m, 2H, H-4’, H-5’), 4.04 (ddd, *J* = 10.2, 3.3, 1.9 Hz, 1H, H-5), 3.94 (t, *J* = 9.3 Hz, 1H, H-3), 3.75 (d, *J* = 11.0 Hz, 1H, H-1’a), 3.72–3.45 (m, 21H, -OC*H*_2_-, H-4, H-2, 2 × C*H*_2_-Cl), 3.42 (dd, *J* = 10.8, 1.8 Hz, 1H, H-6a) ppm; ^13^C NMR δ 138.9, 138.8, 138.4, 138.3, 138.3, 137.9 (C_quat_, 6 × OCH_2_*Ph*), 104.7 (C-2’), 90,2 (C-1), 83.9 (C-3’), 82.6 (C-4’), 81.9 (C-3), 79.8 (C-2), 79.8 (C-5’), 77.5 (C-4), 75.5, 74.8, 73.4, 72.9, 72.3 (5 × O*C*H_2_Ph), 72.7 (C6’), 72.4, 71.4, 71.3, 71.0, 70.8, 70.5, 70.5 (C-7, C-8, C-9, C-7’, C-8’, C9’, C1’), 70.6 (C-5), 69.7 (C-6), 42.8, 42.7 (2 × *C*H_2_Cl) ppm; HRMS (ESI) [M + Na]^+^ calcd for C_62_H_72_O_13_Cl_2_Na, 1117.4248; found, 1117.4211; anal. calcd for C_62_H_72_O_13_Cl_2_ (1096.15): C, 67.94; H, 6.62; Cl, 6.47; found: C, 67.94; H, 6.85; Cl, 6.43.

**1’,2,3,3’,4,4’-Hexa-*O*-benzyl-6,6’-bis[2-(2-iodoethoxy)ethyl]sucrose (10):** A solution of the above bis-chloro derivative **9** (813 mg, 0.74 mmol) in dry acetone (16 mL) containing dry sodium iodide (444.7 mg, 2.97 mmol) was stirred and boiled under reflux for 24 h. After cooling to rt, the precipitate was filtered off and washed with acetone. The combined acetone solutions were concentrated, and the residue was dissolved in CH_2_Cl_2_ (10 mL). The organic phase was washed with water and dried to give **10** (900 mg, 0.70 mmol, 95%) as an oil. [α] = +25.5; ^1^H NMR δ 5.65 (d, *J*_1,2_ = 3.6 Hz, 1H, H-1), 4.90 (d, *J* = 10.9 Hz, 1H, OC*H*_2_Ph), 4.86 (d, *J* = 11.0 Hz, 1H, OC*H*_2_Ph), 4.76 (d, *J* = 10.9 Hz, 1H, OC*H*_2_Ph), 4.68 (d, *J* = 11.4 Hz, 1H, OC*H*_2_Ph), 4,64 (dd, 2H, OC*H*_2_Ph), 4.58–4.54 (m, 4H, OC*H*_2_Ph), 4.43–4.41 (m, 2H, OC*H*_2_Ph, H-3), 3.75 (d, *J* = 11.0 Hz, 1H, H-1’a), 3.71–3.47 (m, 17H, -OC*H*_2_-, H-4, H-2), 3.42 (dd, *J* = 10.8, 1.8 Hz, 1H, H-6a), 3.19–3.16 (m, 2H, C*H*_2_I), 3.15 (t, *J* = 6.9 Hz, 2H, C*H*_2_I) ppm; ^13^C NMR δ 138.9, 138.8, 138.4, 138.3, 138.3, 137.9 (C_quat_, 6 × OCH_2_*Ph*), 104.8 (C-2’), 90,3 (C-1), 83.9 (C-3’), 82.7 (C-4’), 81.9 (C-3), 79.8 (C-2), 79.8 (C-5’), 77.5 (C-4), 75.5, 74.9, 73.4, 72.9, 72.3 (5 × O*C*H_2_Ph), 72.7 (C6’), 72.4, 72.0, 71.9, 70.8, 70.8, 70.1, 70.1 (C-7, C-8, C-9, C-7’, C-8’, C9’, C1’), 70.6 (C-5), 69.7 (C-6), 3.1, 2.9 (2 × *C*H_2_I) ppm; HRMS (ESI) [M + Na]^+^ calcd for C_62_H_72_O_13_I_2_Na, 1301.2960; found, 1301.2955; anal. calcd for C_62_H_72_O_13_I_2_ (1279.05): C, 58.22; H, 5.67; I, 19.84; found: C, 58.12; H, 5.67; I, 19.79.

**Cryptand 11:** To a solution of **10** (258 mg, 0.20 mmol) in acetonitrile (7 mL), powdered potassium carbonate (342 mg, 3.227 mmol, 16 equiv) was added, followed by aza-crown **8** (53 mg, 0.20 mmol, 1 equiv), and the mixture was stirred and boiled under reflux for 24 h (TLC monitoring: dichloromethane/methanol 10:1). The reaction mixture was then cooled to room temperature, diluted with toluene (5 mL), and acetonitrile was removed in vacuum. The remaining toluene solution was passed through a short pad of Celite, concentrated, and the residue was purified by flash chromatography (dichloromethane/methanol 100:0→96:4) to afford derivative **11** (87 mg, 0.07 mmol, 33%) as an oil; ^1^H NMR (acetone-*d*_6_) δ 5.84 (d, *J* = 3.7 Hz, 1H, H-1), 4.95 (d, *J* = 11.2 Hz, 1H, OC*H*_2_Ph), 4.85 (d, *J* = 11.3 Hz, 1H, OC*H*_2_Ph), 4.82–4.77 (m, 4H, OC*H*_2_Ph), 4.72 (d, *J* = 12.1 Hz, 1H, OC*H*_2_Ph), 4.64 (d, *J* = 10.8 Hz, 3H, OC*H*_2_Ph), 4.61 (d, *J* = 11.3 Hz, 1H, OC*H*_2_Ph), 4.58 (d, *J* = 12.0 Hz, 1H, OC*H*_2_Ph), 4.52 (d, *J* = 7.9 Hz, 1H, H-3’), 4.40 (t, *J* = 8.1 Hz, 1H, H-4’), 4.06 (dt, *J* = 10.4, 2.6 Hz, 1H, H-5), 3.97 (ddd, *J* = 8.0, 5.0, 2.7 Hz, 1H, H-5’), 3.92 (t, *J* = 9.3 Hz, 1H, H-3), 3.84 (dd, *J* = 11.2, 2.8 Hz, 1H, H-6’a), 3.80 (dd, *J* = 11.2, 5.1 Hz, 1H, H-6’b), 3.75 (d, *J* = 10.8 Hz, 1H, H-1’a), 3.62 (d, *J* = 10.74 Hz, H-1’b), 3.51 (m, 1H, H-4), 3.48 (dd, *J* = 9.6, 3.7 Hz, 1H, H-2), 3.43 (m, 2H, H-6a, H6b) ppm; ^13^C NMR (acetone-*d*_6_) δ 104.1 (C-2’), 88.7 (C-1), 83.6 (C-3’), 82.0 (C-3), 80.5 (C-4’), 79.6 (C-2), 79.3 (C-5’), 77.7 (C-4), 74.9, 74.2, 73.0, 72.7, 72.0, 71.5 (6 × O*C*H_2_Ph), 72.4 (C-1’), 71.37 (C-7’), 71.06 (C-6’), 70.7 (C-5), 69.9 (C-7), 69.7 (C-6), 58.4, 53.9, 53.6, 53.0, 52.9, 49.8 (6 × *C*H_2_N) ppm; HRMS (ESI) [M + H]^+^ calcd for C_74_H_97_N_2_O_17_, 1285.6787; found, 1285.6803.

**1’,6,6’-Tri-*O*-allyl-2,3,3’,4,4’-penta-*O*-benzylsucrose (17):** To a solution of triol **2** (88 mg; 0.11 mmol) in toluene (3 mL), allyl bromide (56 μL, 0.66 mmole, 6 equiv) was added followed by 50% aq NaOH (3 mL), and the heterogeneous mixture was vigorously stirred at 50 °C for 18 h (TLC monitoring hexane/ethyl acetate 4:1). The mixture was diluted with toluene (10 mL), the organic phase was separated, dried, and the product was purified by chromatography (hexane/ethyl acetate 100:0→70:30) to afford **17** (96 mg, 0.19 mmol, 94%) as an oil. [α] = +37.0; ^1^H NMR δ 5.92–5.80 (m, 3H, H-8, H-*β*, H-8’), 5.69 (d, *J*_1,2_ = 3.5 Hz, 1H, H-1), 5.27–5.20, 5.15–5.11 (m, 6H, H-9a, H-9b, H-γ-a, H-γ-b, H-9’a, H-9’b), 4.42 (d, *J*_3’,4’_ = 7.4 Hz, 1H, H-3’), 4.15 (t, *J*_4’,5’_ = 7.3 Hz, 1H, H-4’), 4.09–4.05 (m, 2H, H-5, H-5’), 4.04–3.95 (m, 4H, H-7a, H-α-a, H-7’a, H-7’b), 3.96 (dd, *J*_2,3_ = 9.6, *J*_3,4_ = 10.0 Hz, 1H, H-3), 3.91 (ddt, *J*_α-a,α-b_ = 12.9, *J*_α-b,β_ = 5.8, *J*_α-b,γ-a_ = *J*_α-b,γ-b_ = 1.4 Hz, 1H, H-α-b), 3.87 (ddt, *J*_7a,7b_ = 12.9, *J*_7b,8_ = 5.8, *J*_7b,9a_ = *J*_7b,9b_ = 1.4 Hz, 1H, H-7b), 3.70 (d, *J*_1’a,1’b_ = 11.0 Hz, 1H, H-1’a), 3.67 (dd, *J*_6’a,6’b_ = 10.4, *J*_5’,6’a_ = 6.4 Hz, 1H, H-6’a), 3.65 (dd, *J*_4,5_ = 10.1 Hz, 1H, H-4), 3.63 (dd, *J*_5’,6’b_ = 4.5 Hz, 1H, H-6’b), 3.526 (dd, *J*_6a,6b_ = 10.5, *J*_5,6a_ = 3.4 Hz, 1H, H-6a), 3.525 (dd, 1H, H-2), 3.48 (d, 1H, H-1’b), 3.41 (dd, *J*_5,6b_ = 1.9 Hz, 1H, H-6b) ppm; ^13^C NMR δ 137.9, 137.7, 137.4, 137.3, 137.3 (C_quat_, 5 × OCH_2_*Ph*), 133.7 (C-9), 133.7 (C-9’), 133.5 (C-γ), 116.2 (C-8), 116.1 (C-β), 115.9 (C-8’), 103.6 (C-2’), 89.1 (C-1), 82.9 (C-3’), 81.7 (C-4’), 80.9 (C-3), 78.8 (C-2), 78.6 (C-5’), 76.6 (C-4), 74.5, 73.8, 72.0, 71.5, 71.2 (5 × O*C*H_2_Ph), 71.4 (C-α), 71.4 (C-7), 71.2 (C-7’), 70.5 (C-6’), 70.0 (C-1’), 69.5 (C-5), 67.4 (C-6) ppm; anal. calcd for C_56_H_64_O_11_ (913.10): C, 73.66; H, 7.06; found: C, 73.74; H, 6.96.

**2,3,3’,4,4’-Penta-*O*-benzyl-1’,6,6’-tri-*O*-(2-methanesulfonyloxyethyl)sucrose (19):** Ozone was bubbled through a cooled solution (−78 °C) of **17** (568 mg, 0.62 mmol) in dichloromethane (40 mL) until the dark blue color persisted (ca 25 min). The cooling bath was removed, methanol (10 mL) was added followed by sodium borohydride (400 mg), and the mixture was stirred for 16 h (TLC monitoring dichloromethane/methanol 20:1). Water (5 mL) was added followed by 10% aq NaOH (5 mL) and brine (5 mL), and the mixture was stirred at rt for another 30 min. The organic phase was separated and the aqueous one was extracted with CH_2_Cl_2_. Combined organic solutions were dried and concentrated, and the crude triol **18** was used in the next step without further purification.

To a solution of the above triol **18** in anh. dichloromethane (10 mL) containing catalytic amounts of DMAP (15 mg), triethylamine (1.3 mL) was added, and the mixture was cooled to −78 °C. Mesyl chloride (0.35 mL) was added dropwise within 5 min, the mixture was allowed to attend room temperature and stirred for 2 h. Then it was partitioned between dichloromethane (20 mL) and water (20 mL), the organic phase was separated, and the aqueous one extracted with CH_2_Cl_2_ (2 × 25 mL). Combined organic solutions were washed with water (2 × 10 mL), dried, concentrated and the product was isolated by chromatography (hexane/ethyl acetate 100:0→25:75) to afford **19** (312 mg, 0.27 mmol, 43% from **17**) as an oil. [α] = +37.2; ^1^H NMR δ 5.59 (d, *J*_1,2_ = 3.5 Hz, 1H, H-1), 4.36 (d, *J*_3’,4’_ = 7.3 Hz, 1H, H-3’), 4.29–4.22 (m, 6H, H-8a, H-8b, H-β-a, H-β-b, H-7’a, H-7’b), 4.05 (t, *J*_4’,5’_ = 7.3 Hz, 1H, H-4’), 3.95 (t, *J*_2,3_ = *J*_3,4_ = 9.3 Hz, 1H, H-3), 3.73–3.63 (m, 7H, H-α-a, H-α-b, H-1’a, H-6’a, H-6’b, H-7’a, H-7’b), 3.59–3.54 (m, 4H, H-4, H-6a, H-7a, H-7b), 3.50 (dd, 1H, H-2), 3.47 (d, *J*_1’a,1’b_ = 11.2 Hz, 1H, H-1’b), 3.41 (dd, *J*_6a,6b_ = 10.8, *J*_5,6b_ = 1.5 Hz, 1H, H-6b), 2.95 (s, 3H, C*H*_3_SO_2_-), 2.93, 2.92 (s, 3H, C*H*_3_SO_2_), 2.92 (s, 3H, C*H*_3_SO_2_) ppm; ^13^C NMR δ 138.6, 138.4, 138.2, 137.9, 137.9 (C_quat_, 5 × OCH_2_*Ph*), 104.3 (C-2’), 90.1 (C-1), 83.4 (C-3’), 81.9 (C-4’), 81.8 (C-3), 79.8 (C-2), 79.6 (C-5’), 77.4 (C-4), 75.5, 74.9, 73.0, 72.8, 72.4 (5 × O*C*H_2_Ph), 72.5 (C-6’), 72.2 (C-1’), 70.6 (C-5), 69.9 (C-6), 69.3 (C-α), 69.2 (C-7), 69.0 (C-7’), 68.9 (C-8’), 68.7 (C-8), 68.6 (C-β), 37.5, 37.5, 37.4 (3 × *C*H_3_SO_2_) ppm; HRMS (ESI) [M + Na]^+^ calcd for C_56_H_70_O_20_S_3_Na, 1181.3520; found, 1181.3524; anal. calcd for C_56_H_70_O_20_S_3_ (1158.34): C, 58.02; H, 6.09; S, 8.30; found: C, 57.74; H, 6.18; S, 8.47.

**Synthesis of cryptand 20:** To a solution of **19** (141 mg; 0.12 mmol) in acetonitrile (10 mL), tripodal amine **15** (55 mg, 0.13 mmol, 1.1 equiv) was added, and the mixture was stirred at 80 °C for 24 h (TLC monitoring dichloromethane/methanol 10:1). After cooling to rt, the mixture was concentrated and the resulting cryptand was purified by chromatography (dichloromethane/methanol 100:0→96:4), to afford **20** (103 mg; 0.08 mmol; 65%) as an amorphous solid. ^1^H NMR (selected signals) δ 5.89 (bs, 1H, H-1), 4.11 (d, *J* = 7.1 Hz, 1H, H3’), 4.04 (m, 2H, H5, H5’), 3.98 (m, H4’, H3), 3.53 (dd, *J*_2,3_ = 9.7, *J*_1,2_ = 3.6 Hz, 1H, H-2), 3.36 (m, H4) ppm; ^13^C NMR δ 138.7, 138.5, 137.9, 137.9, 137.7 (C_quat_, 5 × OCH_2_*Ph*), 104.1 (C-2’), 92.1 (C-1), 84.6 (C3’), 82.1 (C4’), 81.5 (C3), 80.5 (C2), 78.8 (C5’), 78.4 (C4), 70.6 (C5), 75.5, 75.3, 73.2, 72.9, 72.7 (5 × O*C*H_2_Ph), 74.1, 72.06, 72.01, 70.8, 70.3, 68.6 (C-6, C-7, C-6’, C-7’, C-1’, C-α), 60.8, 60.4, 58.6 (3 × N*C*H_2_Ph), 54.5, 53.3, 53.2, 52.6, 52.2, 51.9, 51.5, 48.7 (C-8, C-9, C-10, C-8’, C-9’, C-10’, C-β, C-γ, C-δ) ppm; HRMS (ESI) [M + H]^+^ calcd for C_80_H_95_N_4_O_11_, 1287.6997; found, 1287.7020.

**2,3,3’,4,4’-Penta-*O*-benzyl-1’,6,6’-tri[2-(2-chloroethoxy)ethyl]sucrose (21):** A solution of triol **2** (370 mg, 0.47 mmol) and tetrabutylammonium hydrogensulfate (158.6 g, 0.47 mmol) in bis(2-chloroethyl) ether (1171.7 µL, 9.99 mmol) was vigorously stirred with 50% NaOH solution (1757.6 µL) at room temperature for 3 h. Then CH_2_Cl_2_ (12 mL) and water (12 mL) were added, the organic layer was separated and the aqueous one extracted with CH_2_Cl_2_. Combined organic solutions were washed with water (2 × 10 mL), dried, and concentrated under high vacuum to remove excess of bis(2-chloroethyl) ether. The crude product was purified by chromatography (hexane/ethyl acetate 80:20) to give **21** (303 mg, 0.27 mmol, 58%) as an oil. [α] = +26.6; ^1^H NMR δ 5.67 (d, *J*_1,2_ = 3.6 Hz, 1H, H-1), 4.92 (d, *J* = 10.9 Hz, 1H, OC*H*_2_Ph), 4.87 (d, *J* = 11.0 Hz, 1H, OC*H*_2_Ph), 4.79–4.77 (m, 2H, OC*H*_2_Ph), 4,69 (d, *J* = 11.6 Hz, 1H, OC*H*_2_Ph), 4.65–4.57 (m, 5H, OC*H*_2_Ph), 4.42 (d, *J* = 7.2 Hz, 1H, H-3’), 4.10–4.02 (m, 3H, H-4’, H-5’,H-5), 3.95 (t, *J* = 9.3 Hz, H-3), 3.74 (d, *J* = 11.3 Hz, 1H, H-1’a), 3.70–3.49 (m, 30H, -OC*H*_2_-, H-4, H-2, 3 × C*H*_2_Cl), 3.44 (dd, *J* = 10.8, 1.8 Hz, 1H, H-6a) ppm; ^13^C NMR δ 138.9, 138.8, 138.4, 138.3, 138.3 (C_quat_, 5 × OCH_2_*Ph*), 104.7 (C-2’), 90,2 (C-1), 83.8 (C-3’), 82.5 (C-4’), 81.9 (C-3), 79.8 (C-2), 79.6 (C-5’), 77.5 (C-4), 75.5, 74.8, 73.0, 72.4, 72.4 (5 × O*C*H_2_Ph), 72.1, 71,4, 71.3, 71.3, 71.0, 70.8, 70.8, 70.5, 70.5, 70.4, 60.4 (C-7, C-8, C-9, C-6’, C-7’, C-8’, C9’, C1’, C-α, C-β, C-γ), (70.6 (C-5), 69.8 (C-6), 42.8, 42.8, 42.7 (3 × *C*H_2_Cl) ppm; HRMS (ESI) [M + Na]^+^ calcd for C_59_H_73_O_14_Cl_3_Na, 1133.3964; found, 1133.3960; anal. calcd for C_59_H_73_O_14_Cl_3_ (1112.57): C, 63.69; H, 6.57; Cl, 9.56; found: C, 63.69; H, 6.61; Cl, 9.56.

**2,3,3’,4,4’-Penta-*O*-benzyl-1’,6,6’-tri[2-(2-iodoethoxy)ethyl]sucrose (22):** A mixture of bis-chloro derivative **21** (235 mg, 0.21 mmol) and dry NaI (569.9 mg, 3.80 mmol) in dry acetone (7 mL) was stirred and boiled under reflux. After 24 h another portion of sodium iodide (190 mg, 1.27 mmol) was added and stirring under reflux was continued for 24 hours. After cooling, the precipitate was filtered off and washed with acetone. The combined organic solutions were concentrated and the residue dissolved in CH_2_Cl_2_ (5 mL). The organic phase was washed with water and dried to give **22** (279 mg, 0.20 mmol, 95%) as an oil. [α] = +20.9; ^1^HNMR δ 5.66 (d, *J*_1,2_ = 3.6 Hz, 1H, H-1), 4.91 (d, *J* = 10.9 Hz, 1H, OC*H*_2_Ph), 4.87 (d, *J* = 11.0 Hz, 1H, OC*H*_2_Ph), 4.97–4.76 (m, 2H, OC*H*_2_Ph), 4.69 (d, *J* = 11.6 Hz, 1H, OC*H*_2_Ph), 4.65–4.54 (m, 5H, OC*H*_2_Ph), 4.42 (d, *J*_3`,4`_ = 6.9 Hz, 1H, H-3’), 4.13–4.02 (m, 3H, H-4`, H-5`, H-5), 3.95 (t, *J* = 9.3 Hz, 1H, H-3), 3.76–3.47 (m, 21H, -OC*H*_2_-, H-4, H-2), 3.44 (dd, *J* = 10.9, *J* = 1.8 Hz, 1H, H-6), 3.19–3.13 (m, 6H, 3×C*H*_2_I) ppm; ^13^C NMR δ 138.9, 138.8, 138.4, 138.3, 138.3 (C_quat_, 5 × OCH_2_*Ph*), 104.7 (C-2’), 90,2 (C-1), 83.8 (C-3’), 82.6 (C-4’), 81.9 (C-3), 79.8 (C-2), 79.6 (C-5’), 77.5 (C-4), 75.5, 74.9, 73.0, 72.5, 72.4 (5 × O*C*H_2_Ph), 72.8, 72.1, 72.0, 71.9, 71.9, 71.0, 70.8, 70.8, 70.1, 70.1, 70.0 (C-7, C-8, C-9, C-6’, C-7’, C-8’, C9’, C1’, C-α, C-β, C-γ), 70.6 (C-5), 69.8 (C-6), 3.1, 3.0, 2.9 (3 × *C*H_2_I) ppm; HRMS (ESI) [M + Na]^+^ calcd for C_59_H_73_O_14_I_3_Na, 1409.2032; found, 1409.2017; anal. calcd for C_59_H_73_O_14_I_3_ (1386.93): C, 51.09; H, 5.31; I, 27.45; found: C, 51.09; H, 5.31; I, 27.32.

**Synthesis of cryptand 23:** To a solution of **22** (118 mg, 0.09 mmol) in acetonitrile (15 mL), powdered potassium carbonate (270 mg, 2.55 mmol, 30 equiv) was added, followed by amine **15** (40 mg, 0.09 mmol, 1.1 equiv), and the mixture was stirred and boiled under reflux for 24 h (TLC monitoring: dichloromethane/methanol 10:1). The mixture was cooled to room temperature, diluted with toluene (15 mL), and acetonitrile was removed in vacuum. The toluene solution was then filtered through a short pad of Celite, the filtrate was concentrated, and the residue was purified by flash chromatography (dichloromethane/methanol 100:0→93:7) to afford derivative **23** (55 mg, 0.04 mmol, 45.5%) as an oil. ^1^H NMR (acetone-*d*_6_) δ 5.76 (d, *J*_1,2_ = 3.55 Hz, 1H, H-1), 4.99 (d, *J* = 11.01 Hz, 1H, OC*H_2_*Ph), 4.88 (d, *J* = 11.38, 1H, OC*H*_2_Ph), 4.91 (d, *J* = 10.98 Hz, 1H, OC*H*_2_Ph), 4.81 (d, *J* = 11.05 Hz, 1H, OC*H*_2_Ph), 4.71–4.59 (m, 5H, OC*H*_2_Ph), 4.44 (m, 2H, H-3’, OC*H*_2_Ph), 4.05–4.00 (m, 3H, H-4`, H-5`, H-5), 3.96 (t, *J* = 9.24 Hz, 1H, H-3), 3.87 (d, *J* = 11.04 Hz, 1H, -OC*H*_2_-), 3.82 (dd, -OC*H*_2_-), 3.70–3.36 (m, 32H, -OC*H*_2_-, -NC*H*_2_-, H-2, H-4), 3.21–2.54 (m, 16H, -OC*H*_2_-, -NC*H*_2_-) ppm; ^13^C NMR (acetone-*d*_6_) δ 138.6, 138.6, 138.4, 138.2, 138.1, 138.0, 137.9, 137.9 (C_quat_, 8 × CH_2_*Ph*), 105.0 (C-2’), 91.7 (C-1), 83.3 (C-3’), 82.8 (C-4’), 81.6 (C-3), 80.2 (C-2), 80.2 (C-5’), 78.3 (C-4), 75.5, 75.3, 73.1, 72.9, 72.5 (5 × O*C*H_2_Ph), 70.6 (C-5), 59.3, 59.3, 57.3 (3 × N*C*H_2_Ph) 51.9, 51.2, 50.2, 49.5, 49.2, 49.2, 48.1, 48.1, 47.8 (9 × N*C*H_2_-) ppm; HRMS (ESI) [M + Na]^+^ calcd for C_86_H_107_N_4_O_14_Na, 1419.7784, found, 1419.7803.

## Supporting Information

File 1 Copies of NMR spectra.
