# Supplementary material for: First synthesis of cryptands with sucrose scaffold
Source: Beilstein J Org Chem. 2019 Jan 23;15:210–7. doi: 10.3762/bjoc.15.20 (PMC6350891; doi:10.3762/bjoc.15.20)
Supplement: File 1 — Copies of NMR spectra. [file Beilstein_J_Org_Chem-15-210-s001.pdf]

BEILSTEIN JOURNAL OF ORGANIC CHEMISTRY

Supporting Information

for

First synthesis of cryptands with sucrose scaffold

Patrycja Sokołowska, Michał Kowalski and Sławomir Jarosz

Beilstein J. Org. Chem. **2019,** *15,* 210–217. doi:10.3762/bjoc.15.20

Copies of NMR spectra

License and Terms: This is a supporting information file under the terms of the Creative Commons Attribution License (http://creativecommons.org/licenses/by/4.0). Please note that the reuse, redistribution and reproduction in particular requires that the authors and source are credited.
The license is subject to the *Beilstein Journal of Organic Chemistry* terms and conditions: (https://www.beilstein-journals.org/bjoc)

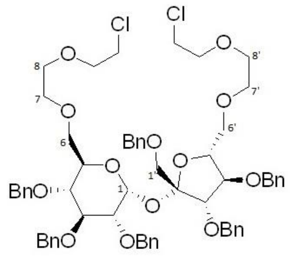

9

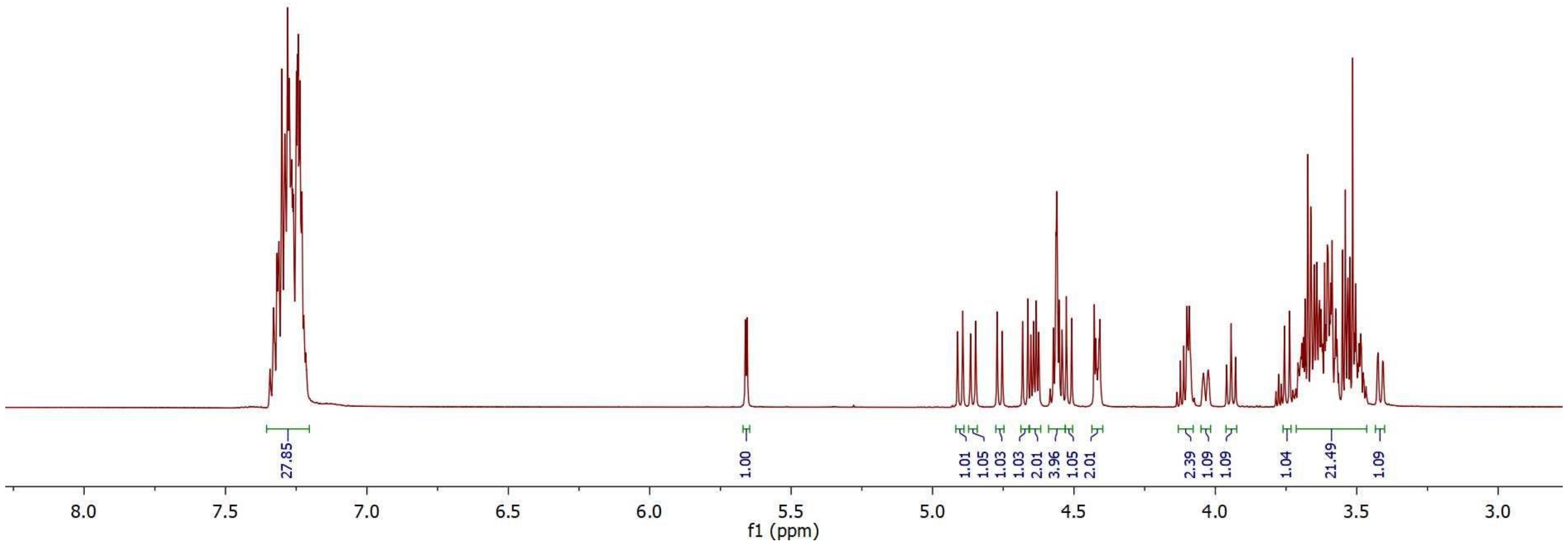

Figure S1 ^{1}H NMR spectrum of compound **9** ($CDCl_3$).

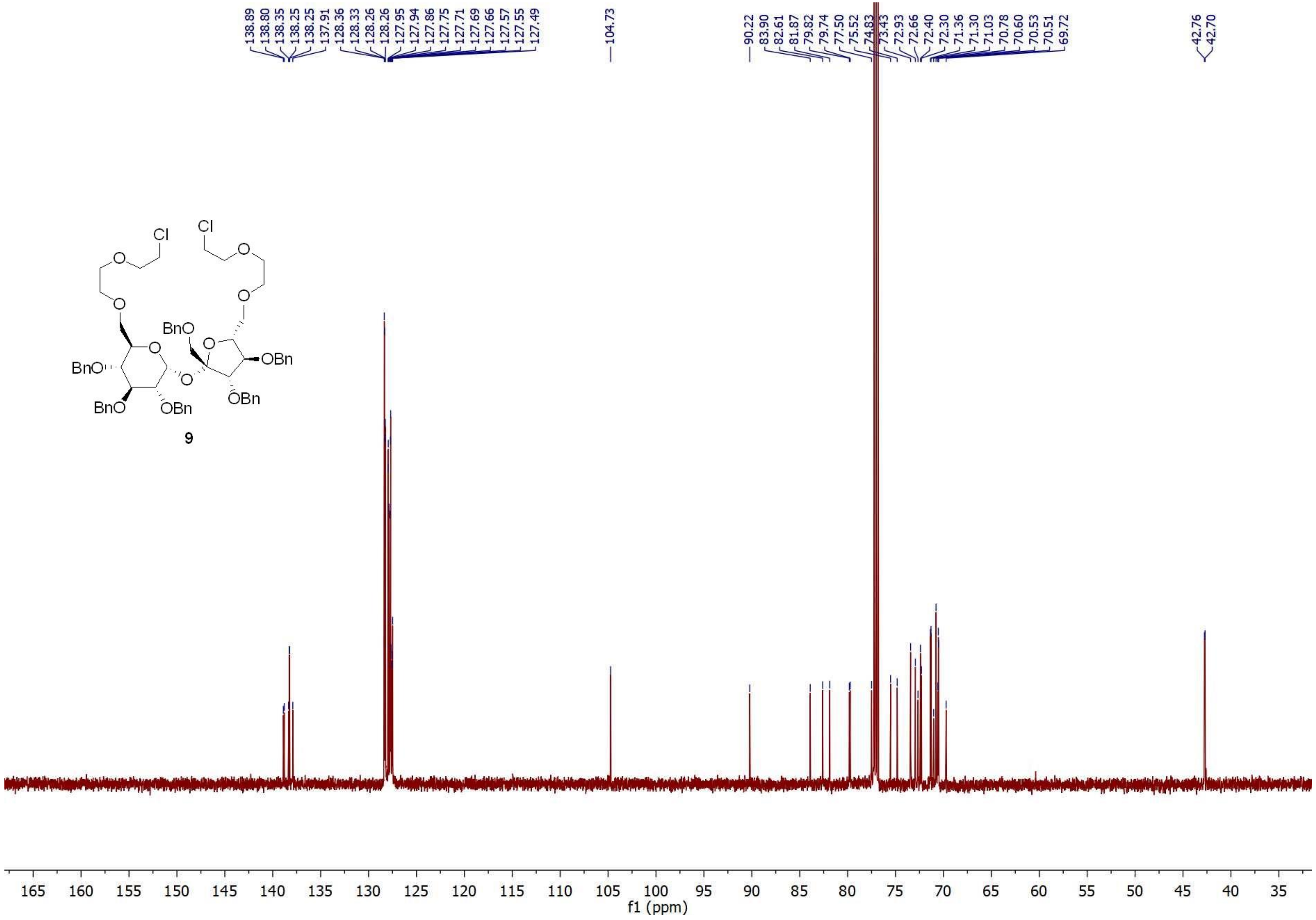

Figure S2 ^{13}C NMR spectrum of compound **9** ($CDCl_3$).

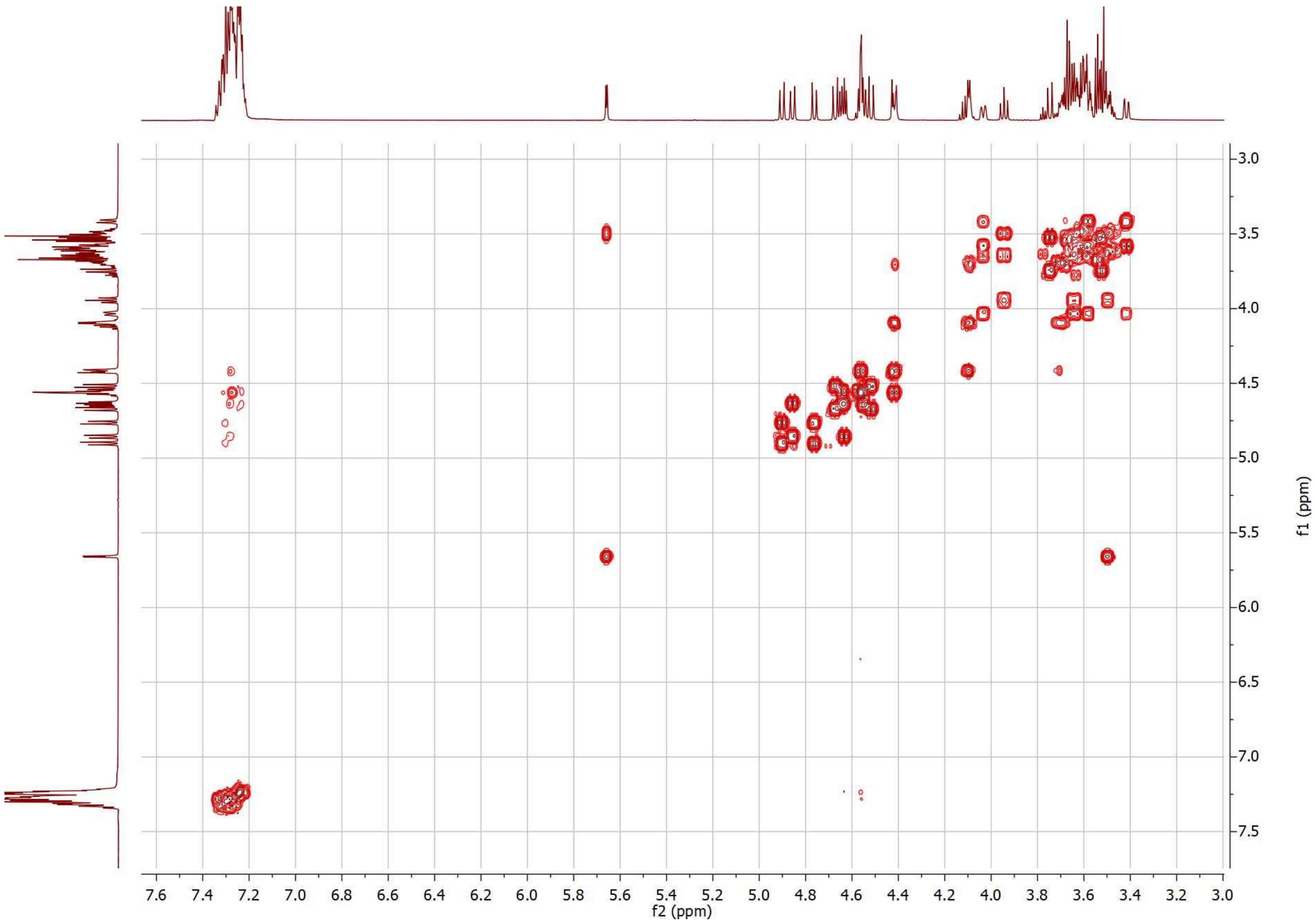

Figure S3 gCOSY spectrum of compound **9**.

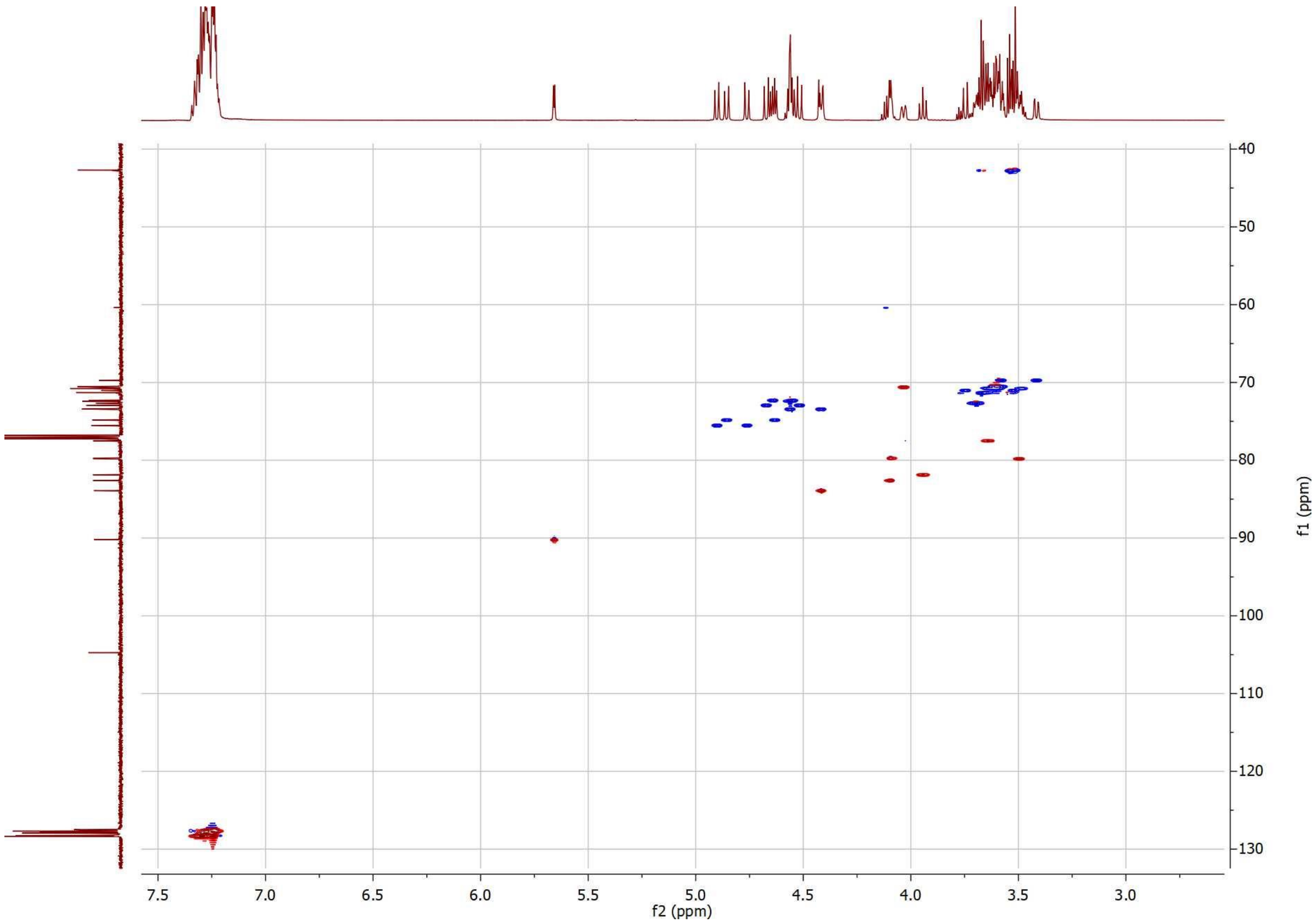

Figure S4 gHSQCAD spectrum of compound **9**.

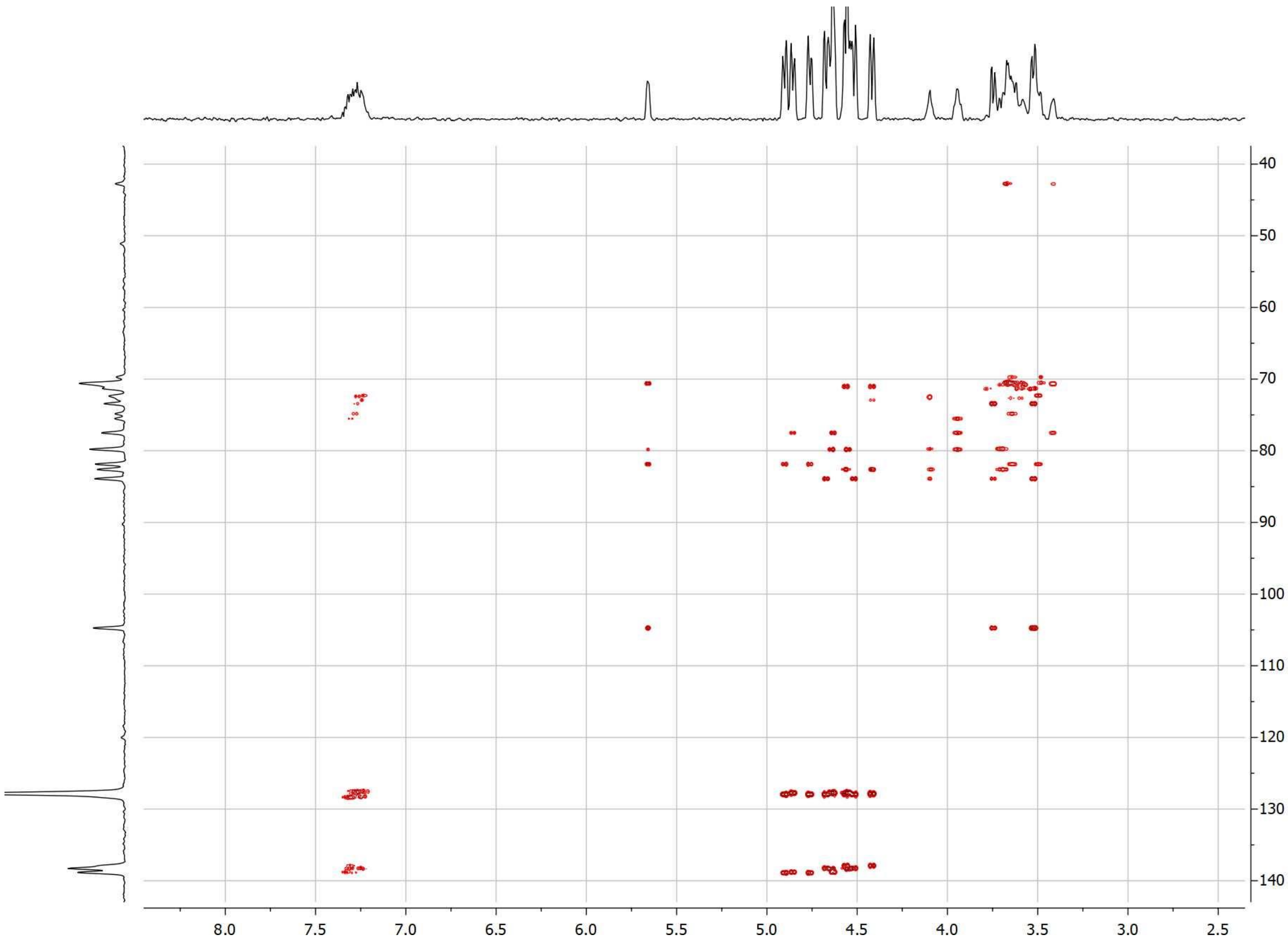

Figure S5 gHMBCAD spectrum of compound **9**.

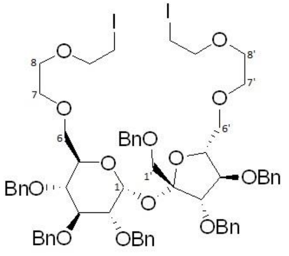

10

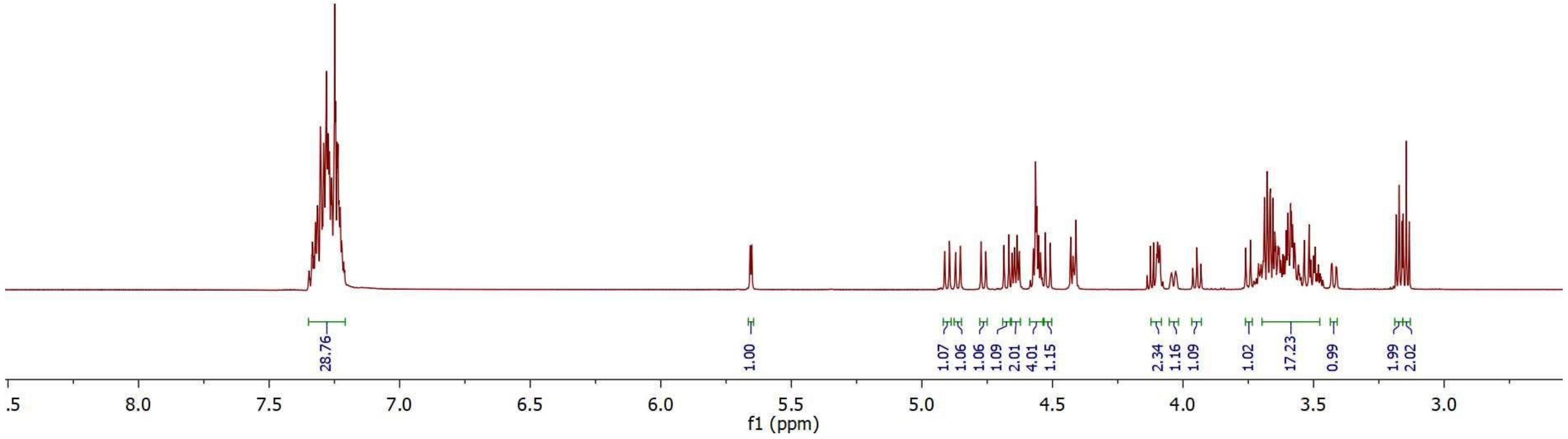

Figure S6 ^{1}H NMR spectrum of compound **10** ($CDCl_3$).

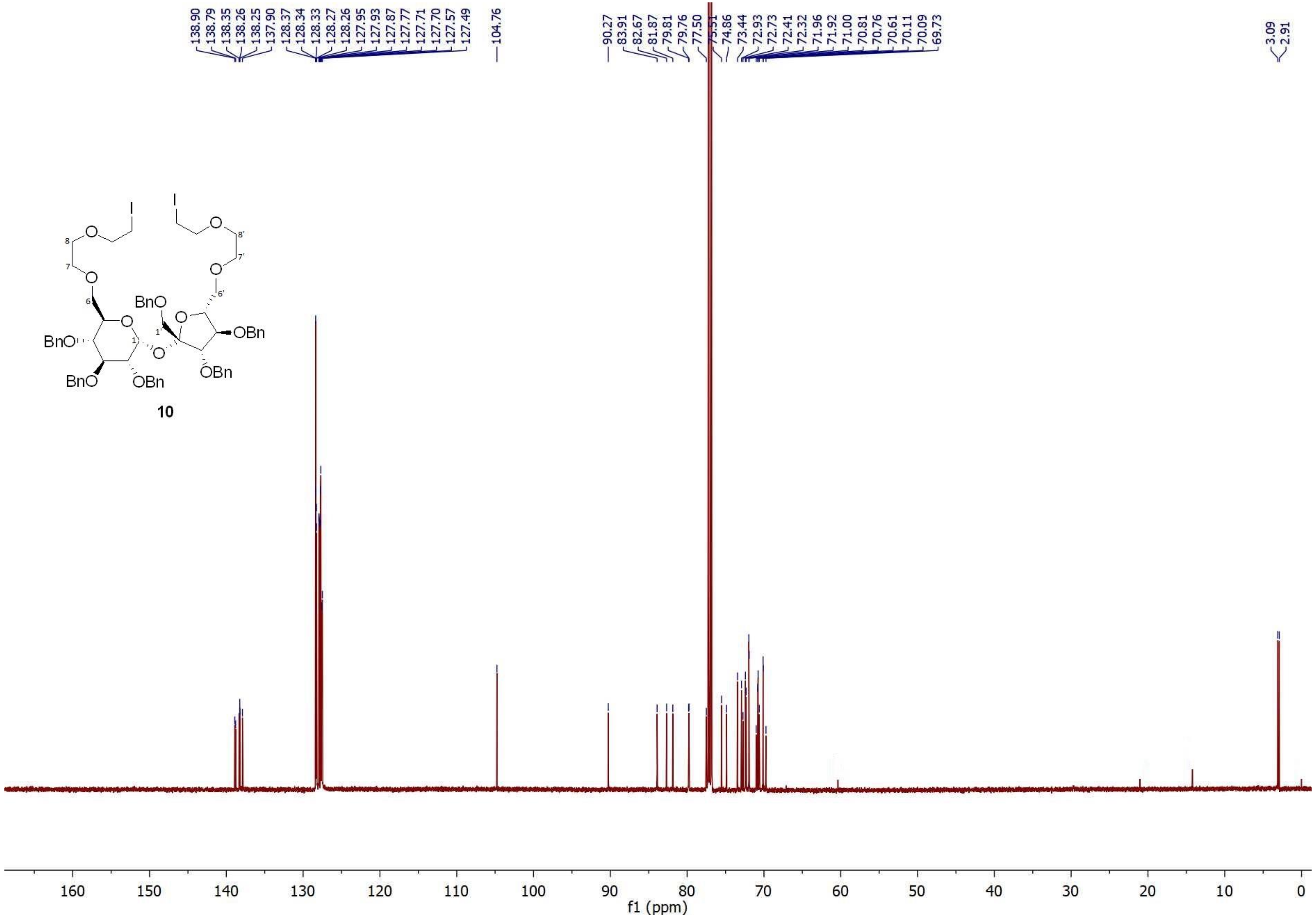

Figure S7 ^{13}C NMR spectrum of compound **10** ($CDCl_3$).

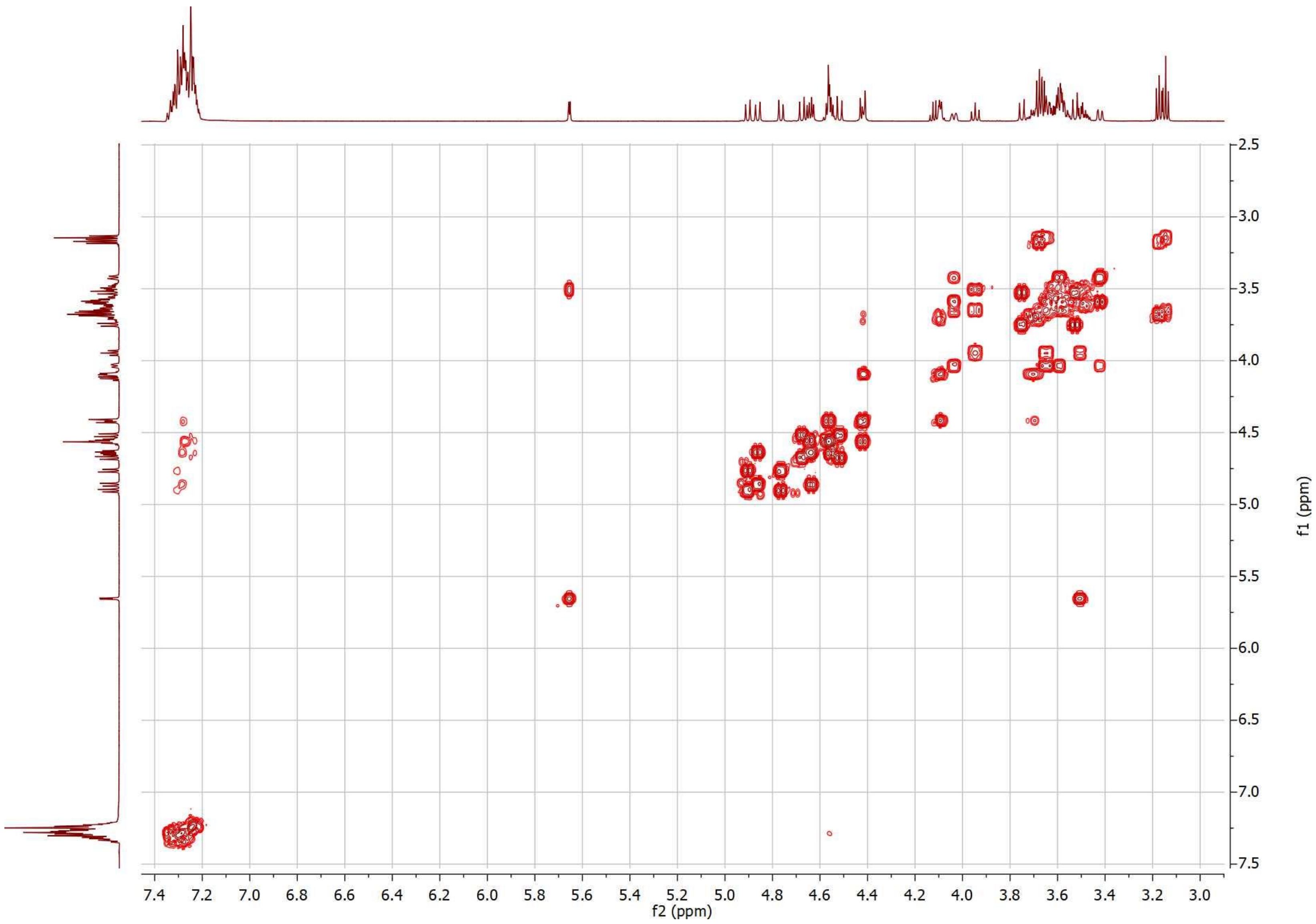

Figure S8 gCOSY spectrum of compound **10**.

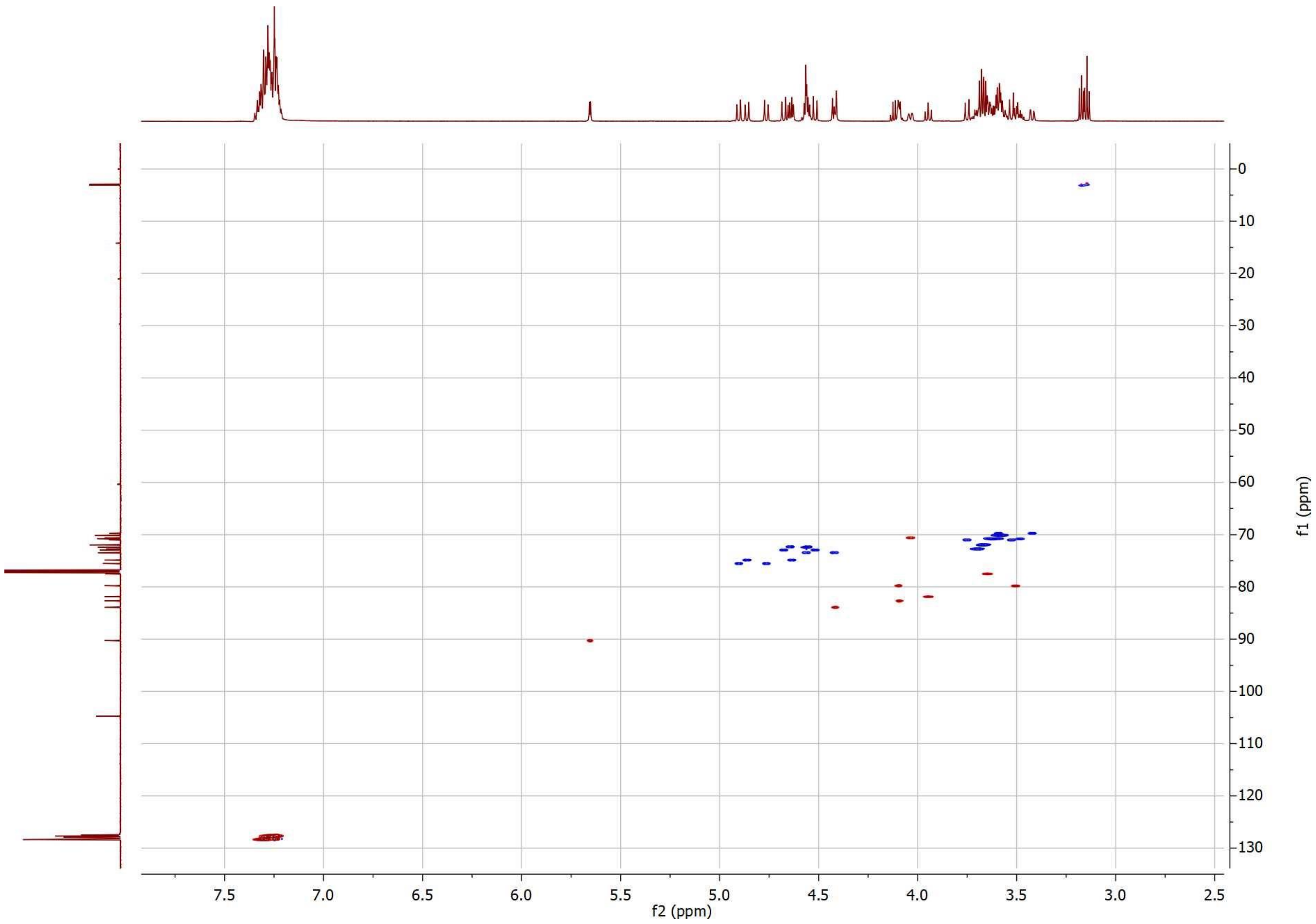

Figure S9 gHSQCAD spectrum of compound **10**

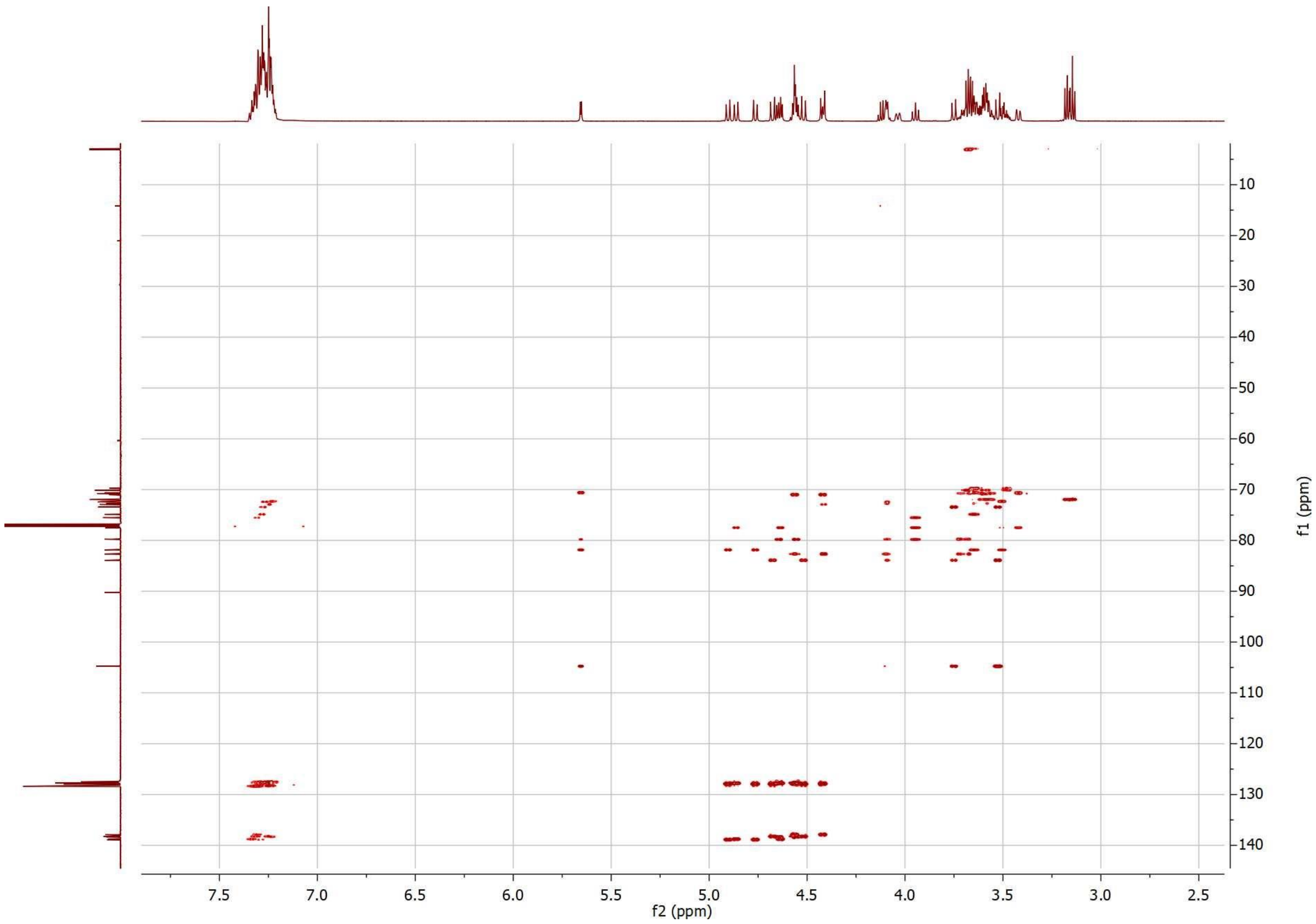

Figure S10 gHMBCAD spectrum of compound **10**.

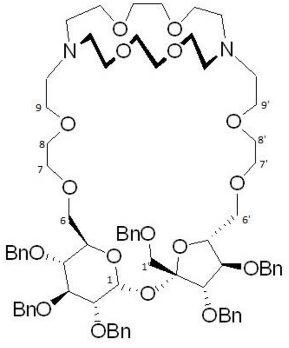

11

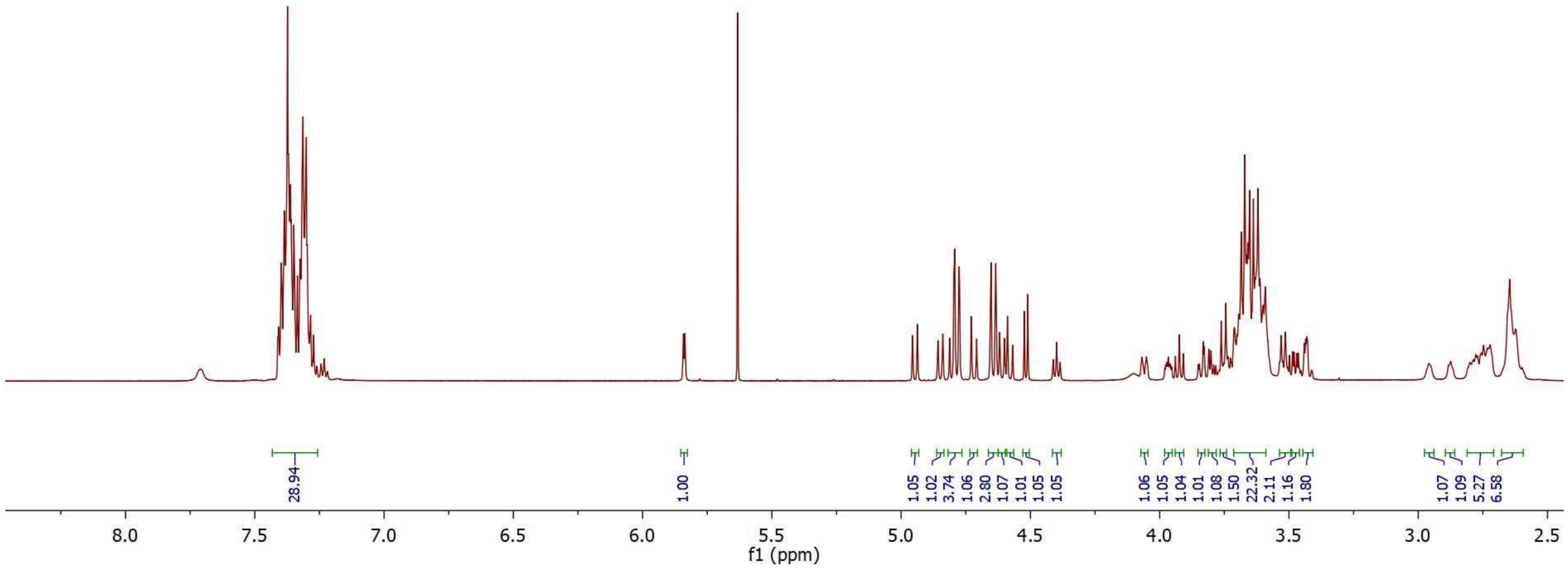

Figure S11 ^{1}H NMR spectrum of compound **11** (acetone-d_6).

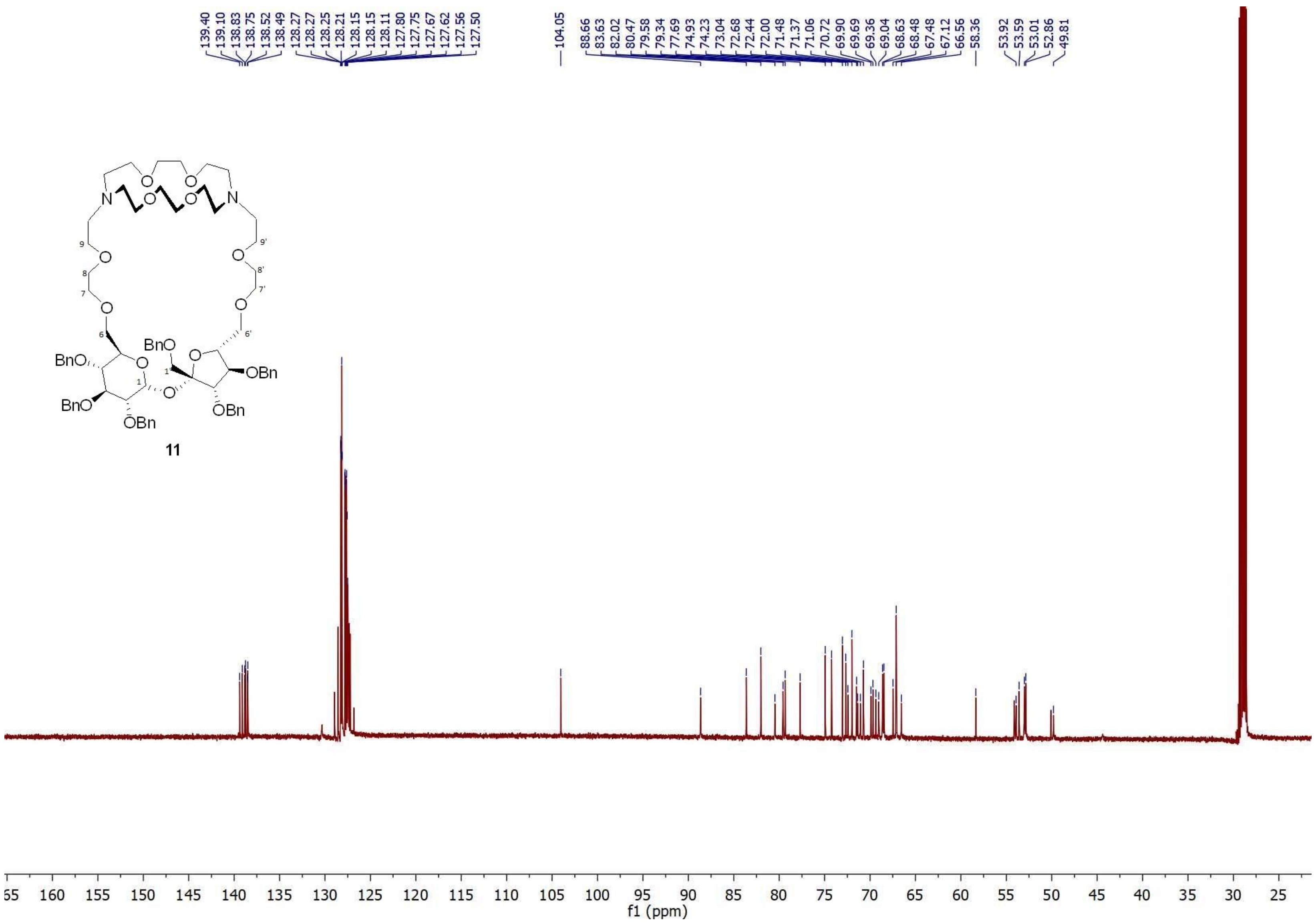

Figure S12 ^{13}C NMR spectrum of compound **11** (acetone-d_6).

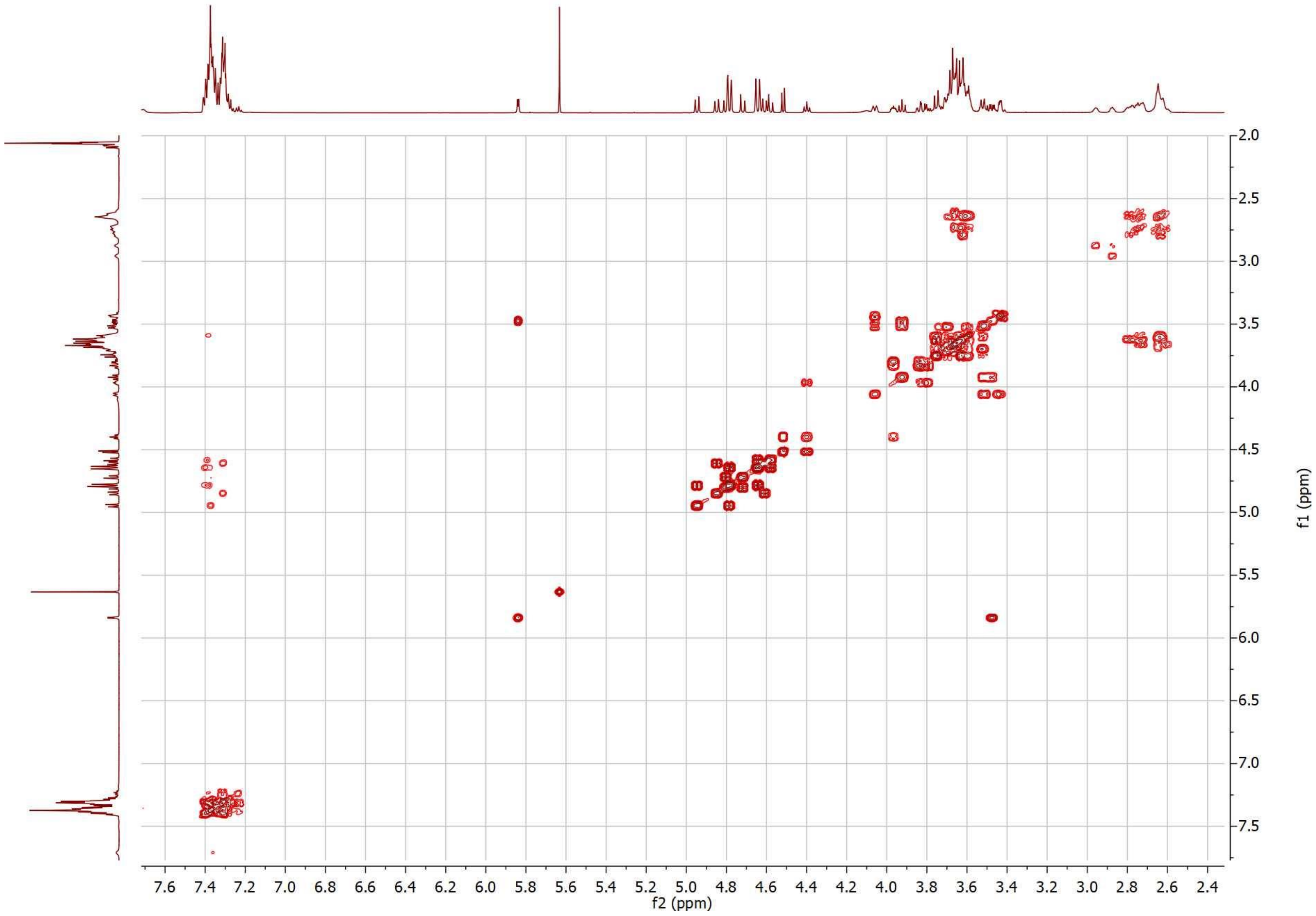

Figure S13 gCOSY spectrum of compound **11**.

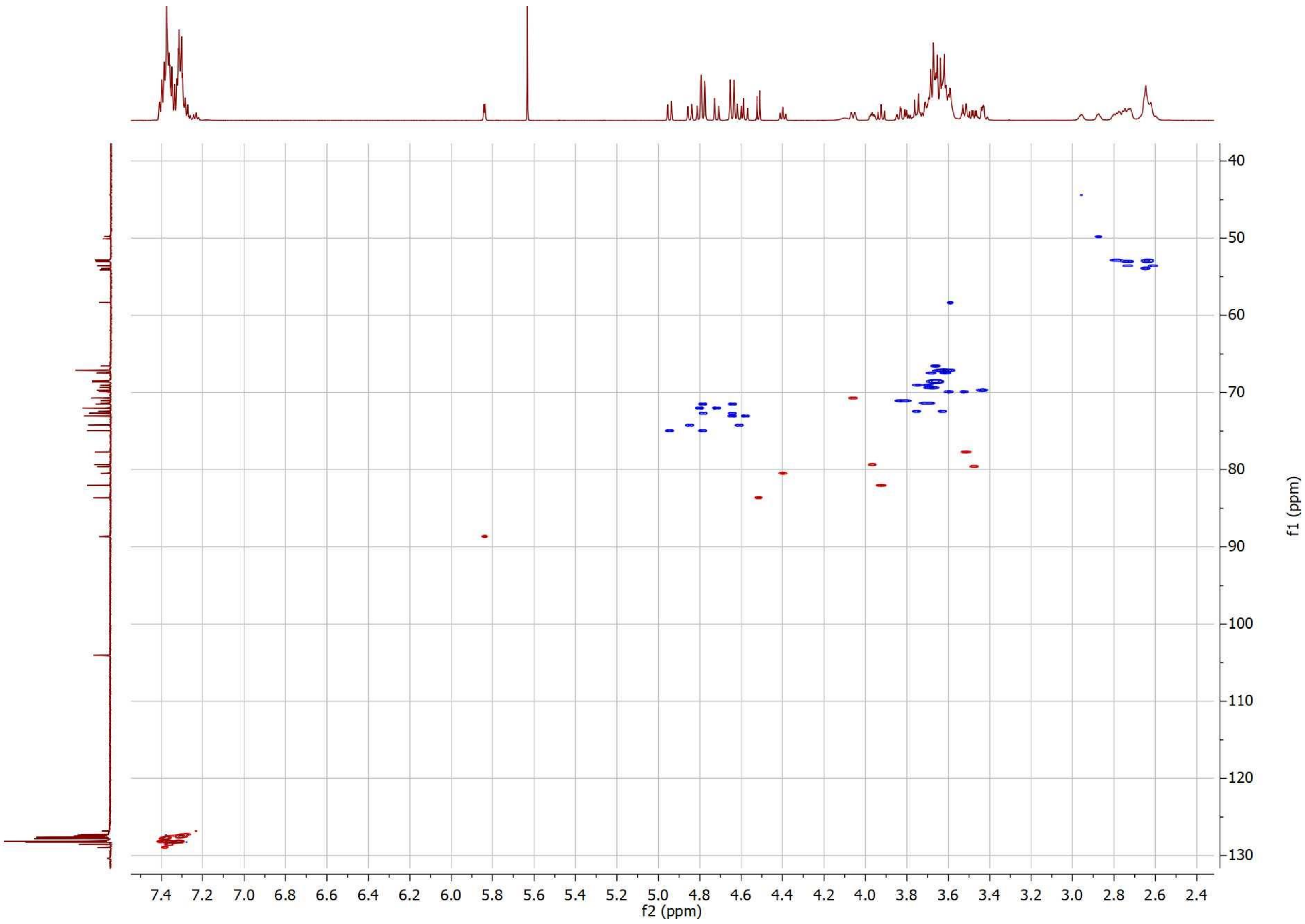

Figure S14 gHSQCAD spectrum of compound **11**.

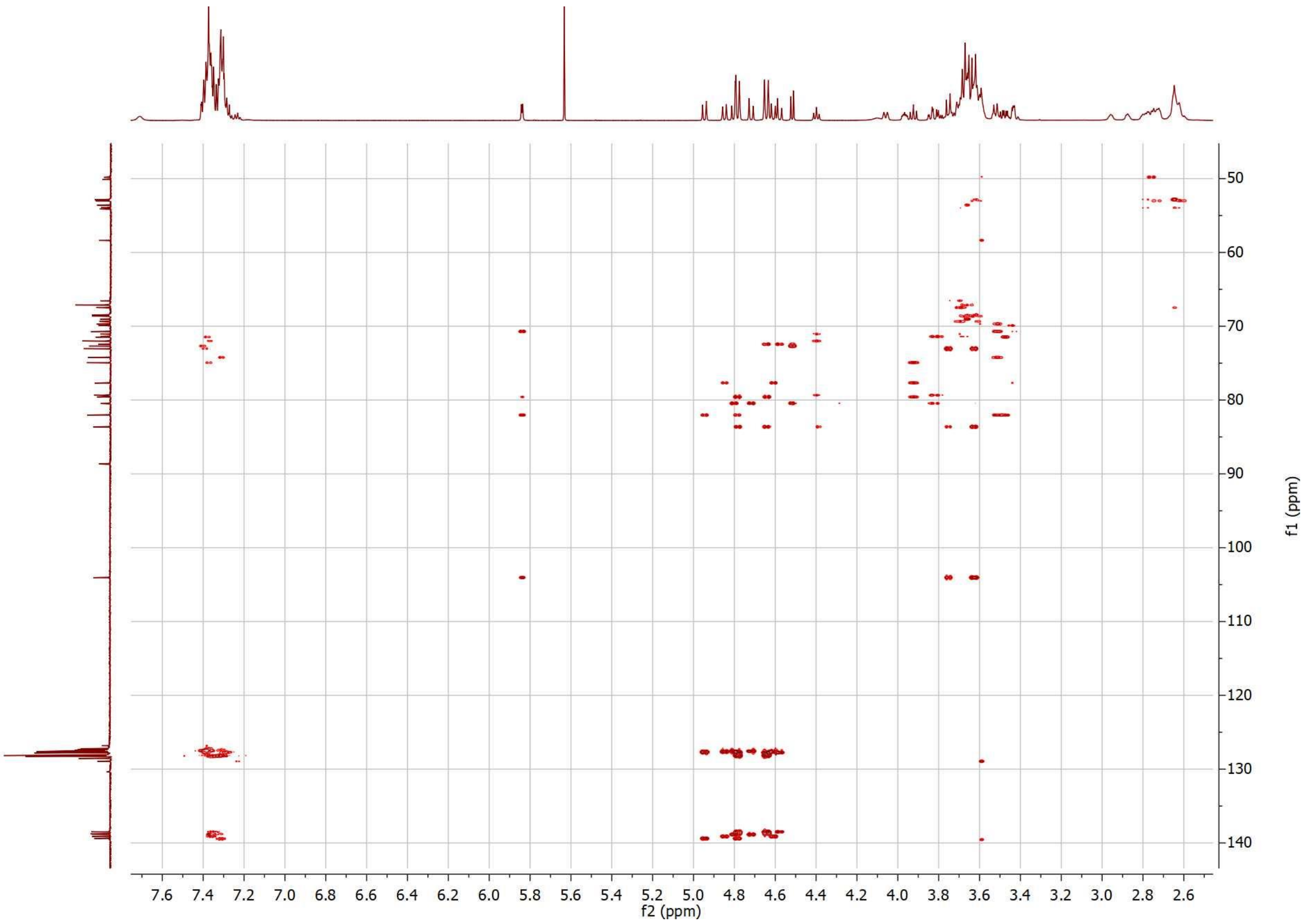

Figure S15 gHMBCAD spectrum of compound **11**.

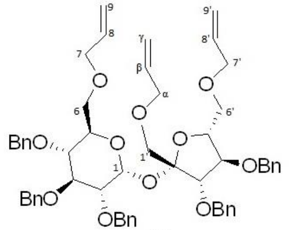

17

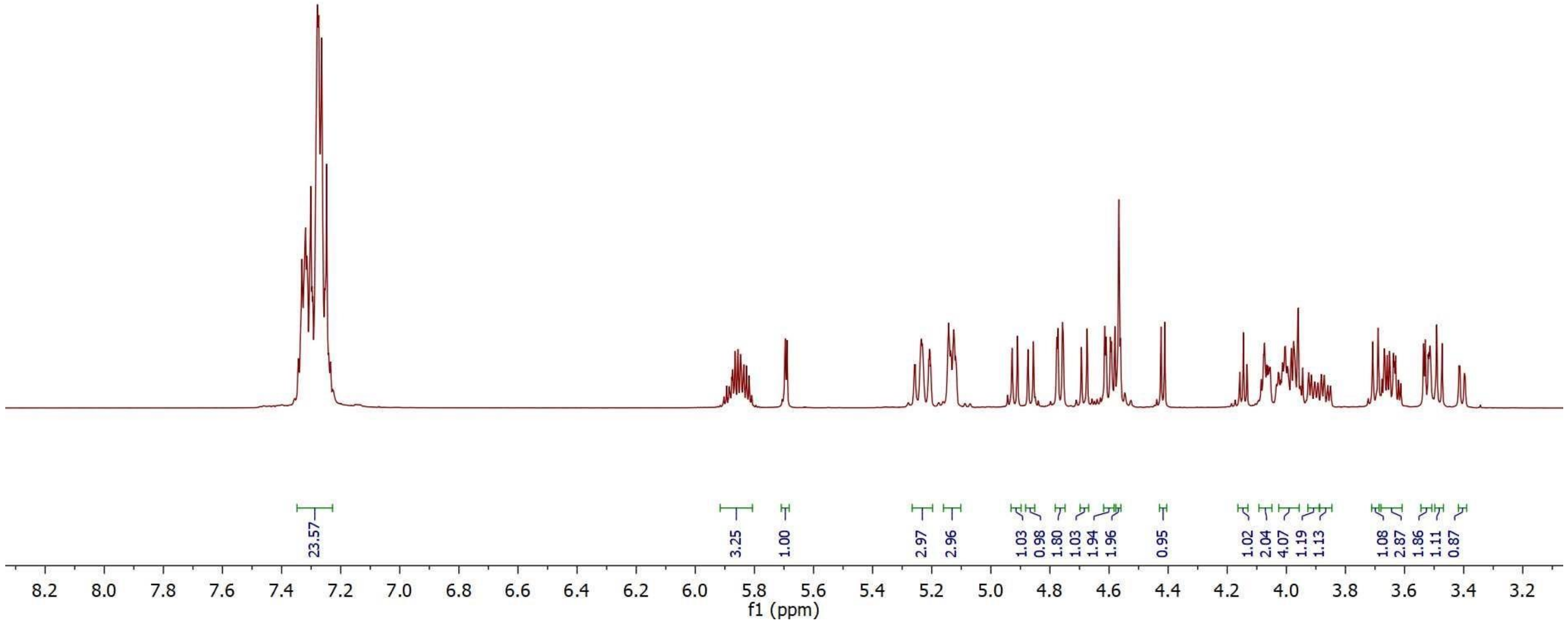

Figure S16 ^{1}H NMR spectrum of compound **17** ($CDCl_3$).

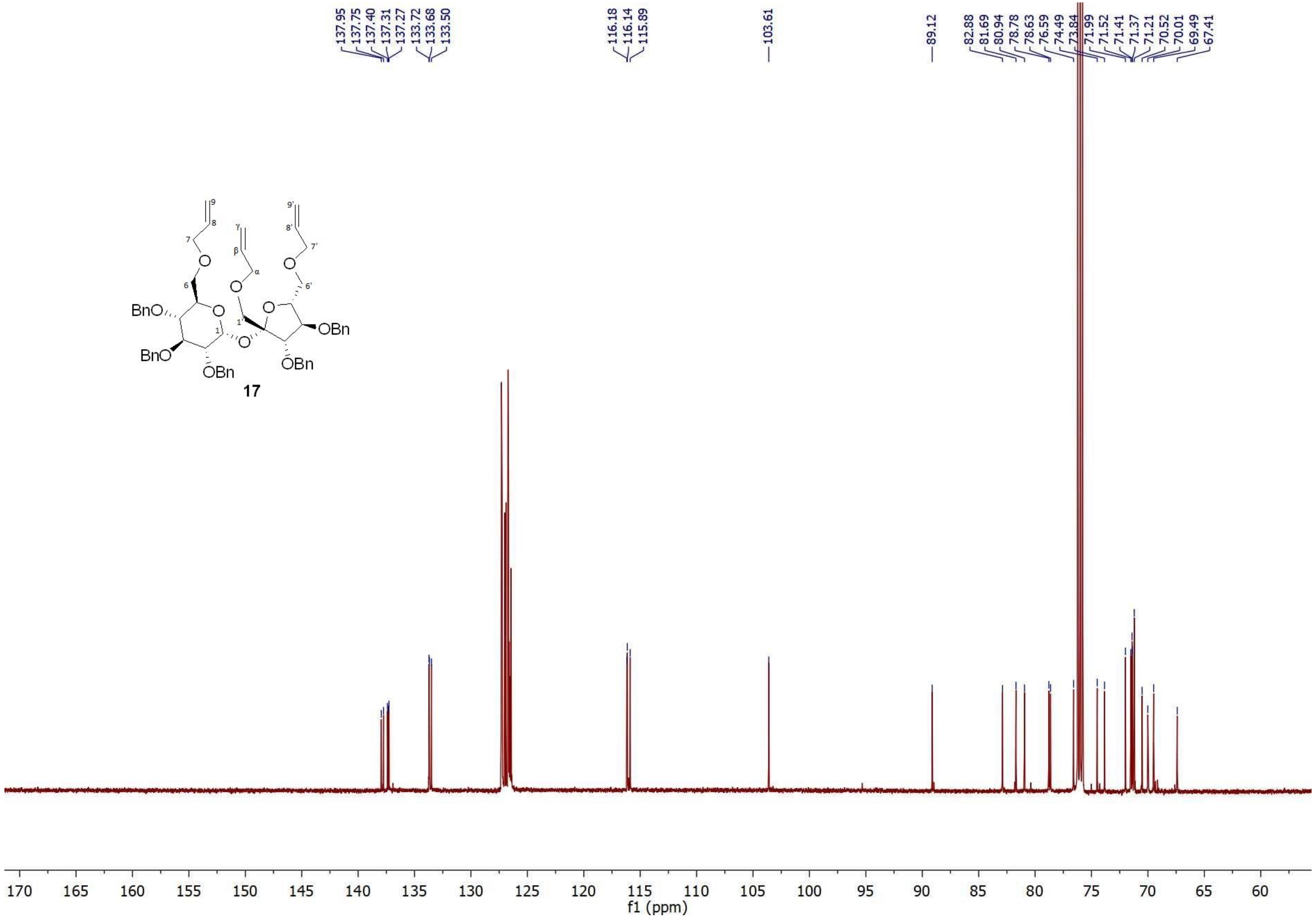

Figure S17 ^{13}C NMR spectrum of compound **17** ($CDCl_3$).

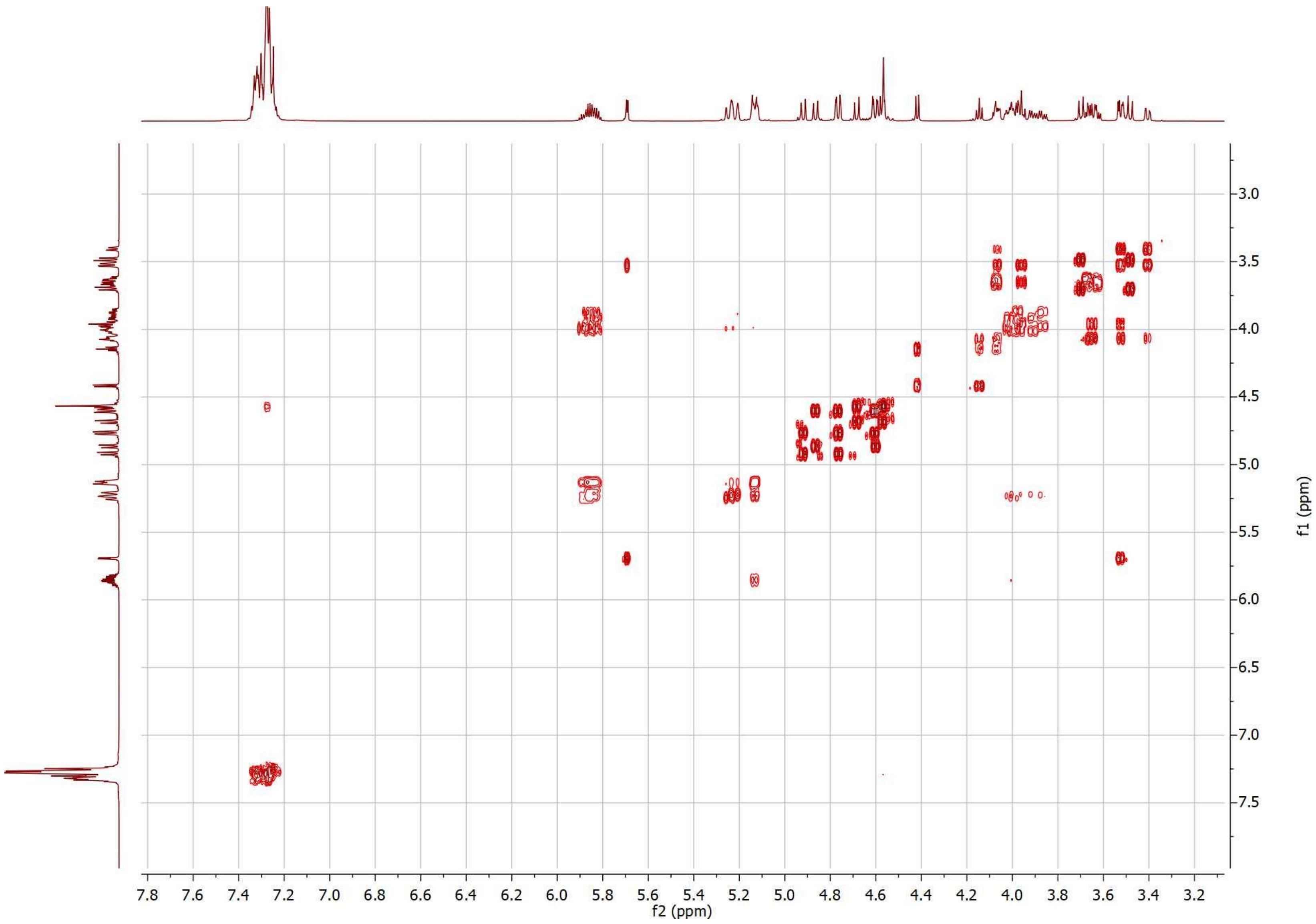

Figure S18 gCOSY spectrum of compound **17**.

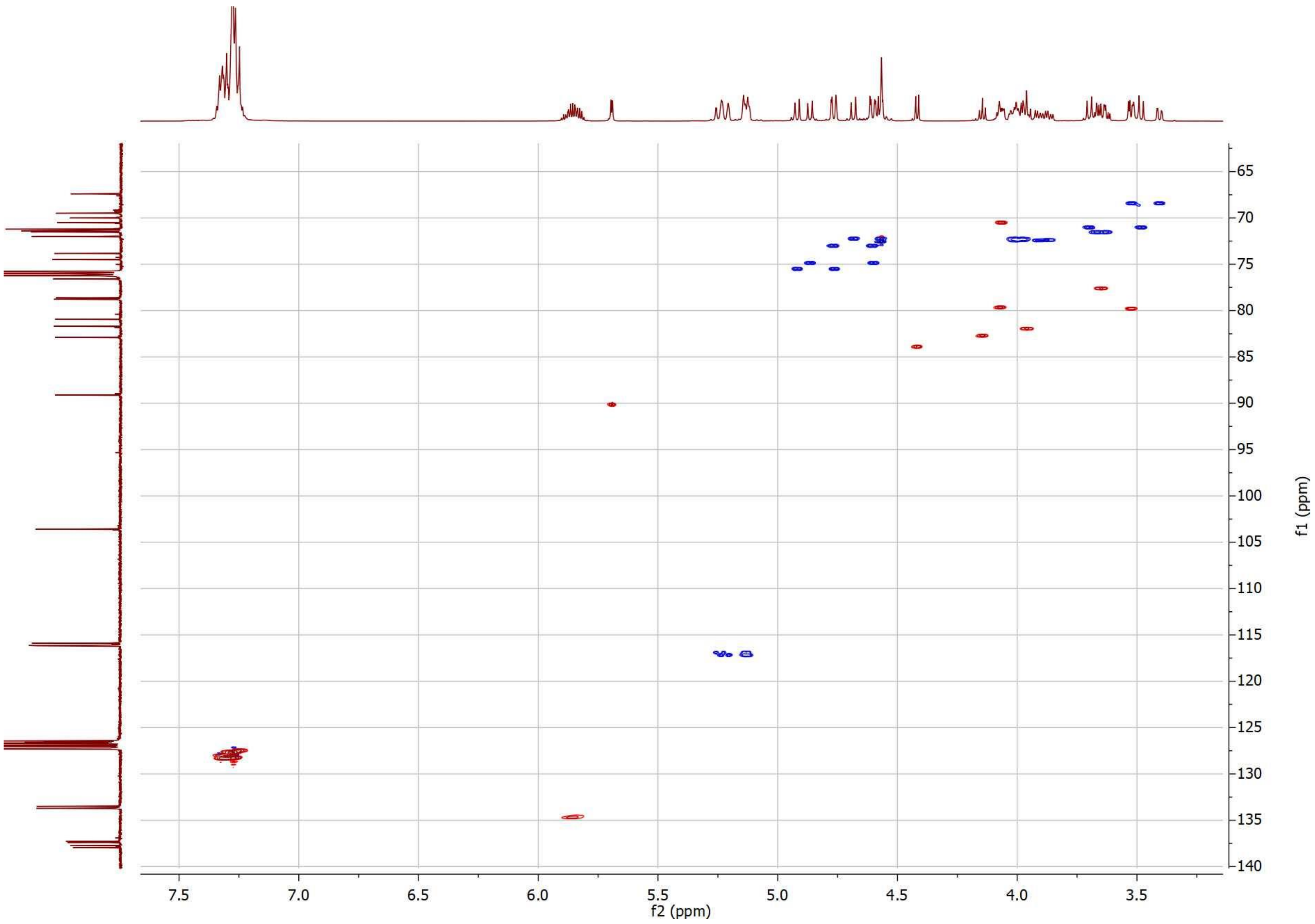

Figure S19 gHSQCAD spectrum of compound **17**.

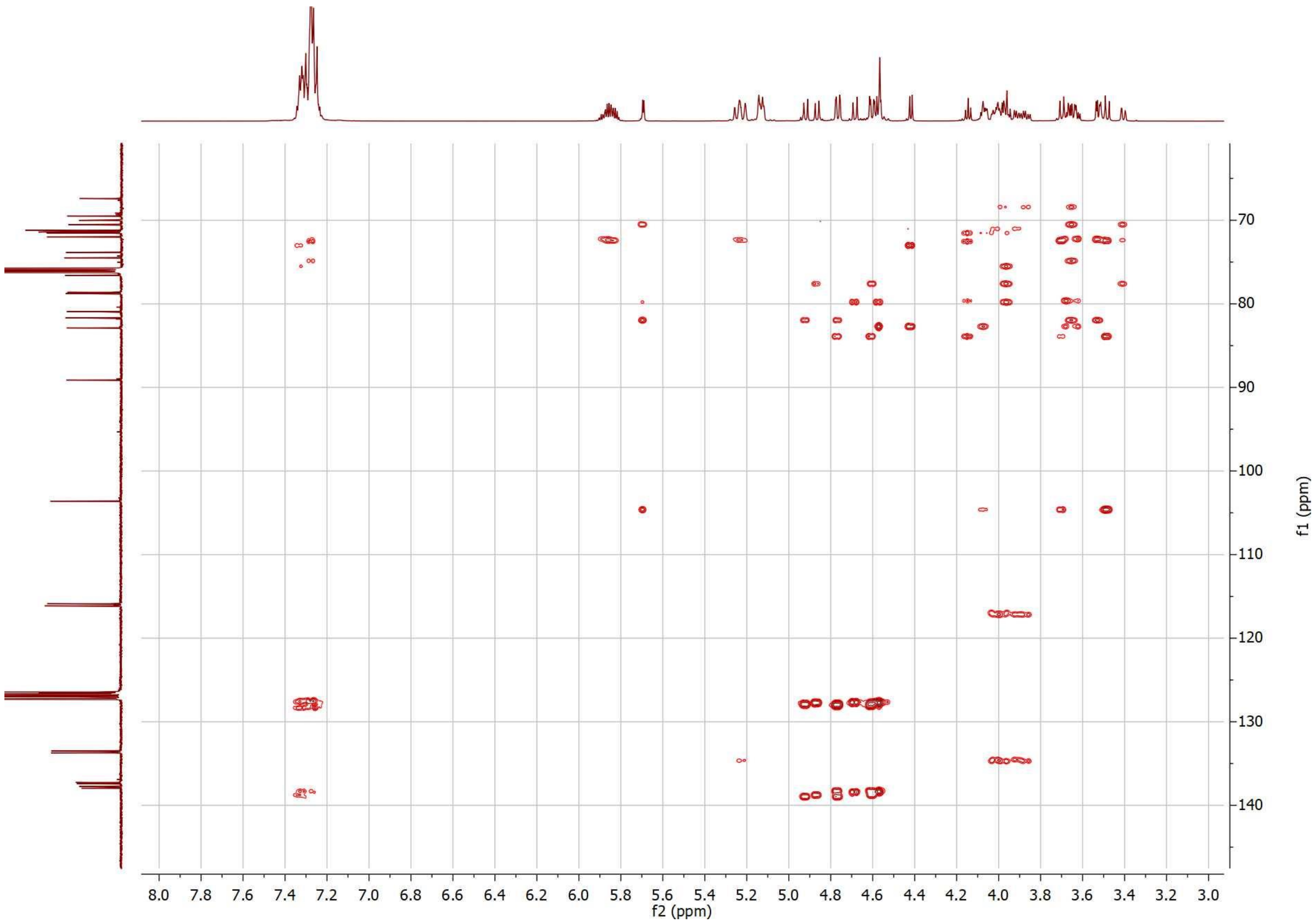

Figure S20 gHMBCAD spectrum of compound **17**.

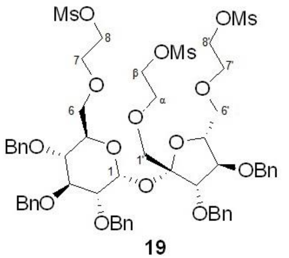

19

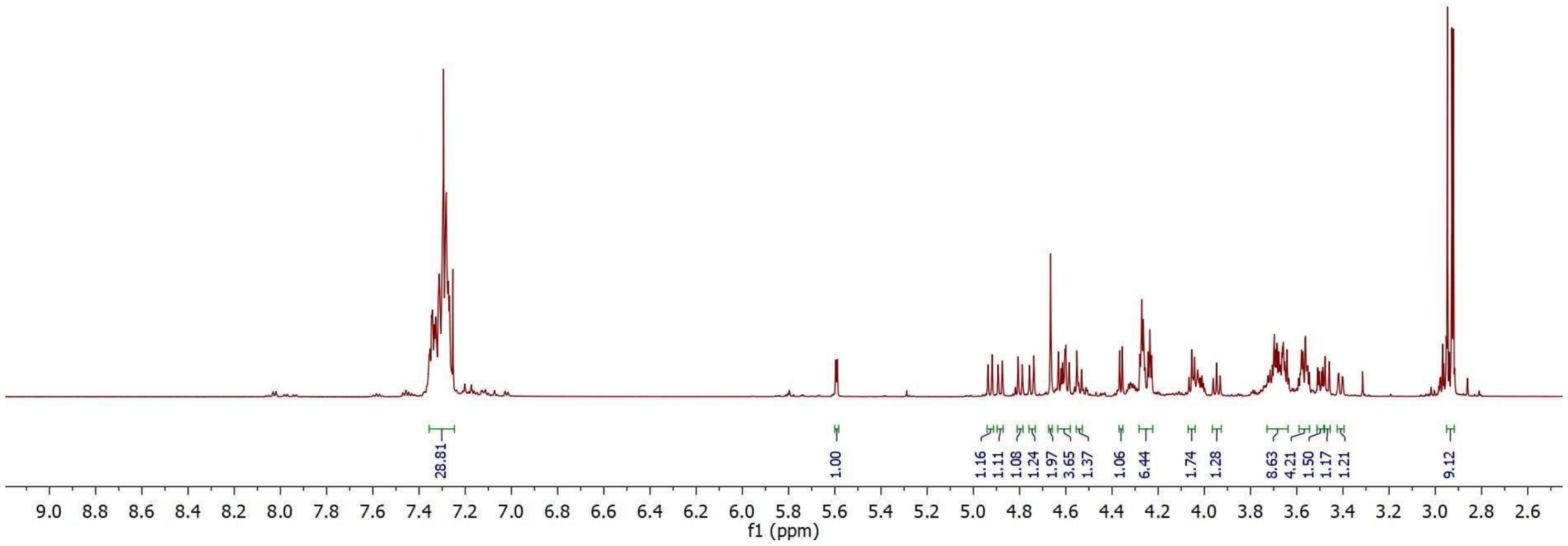

Figure S21 ^{1}H NMR spectrum of compound **19** ($CDCl_3$).

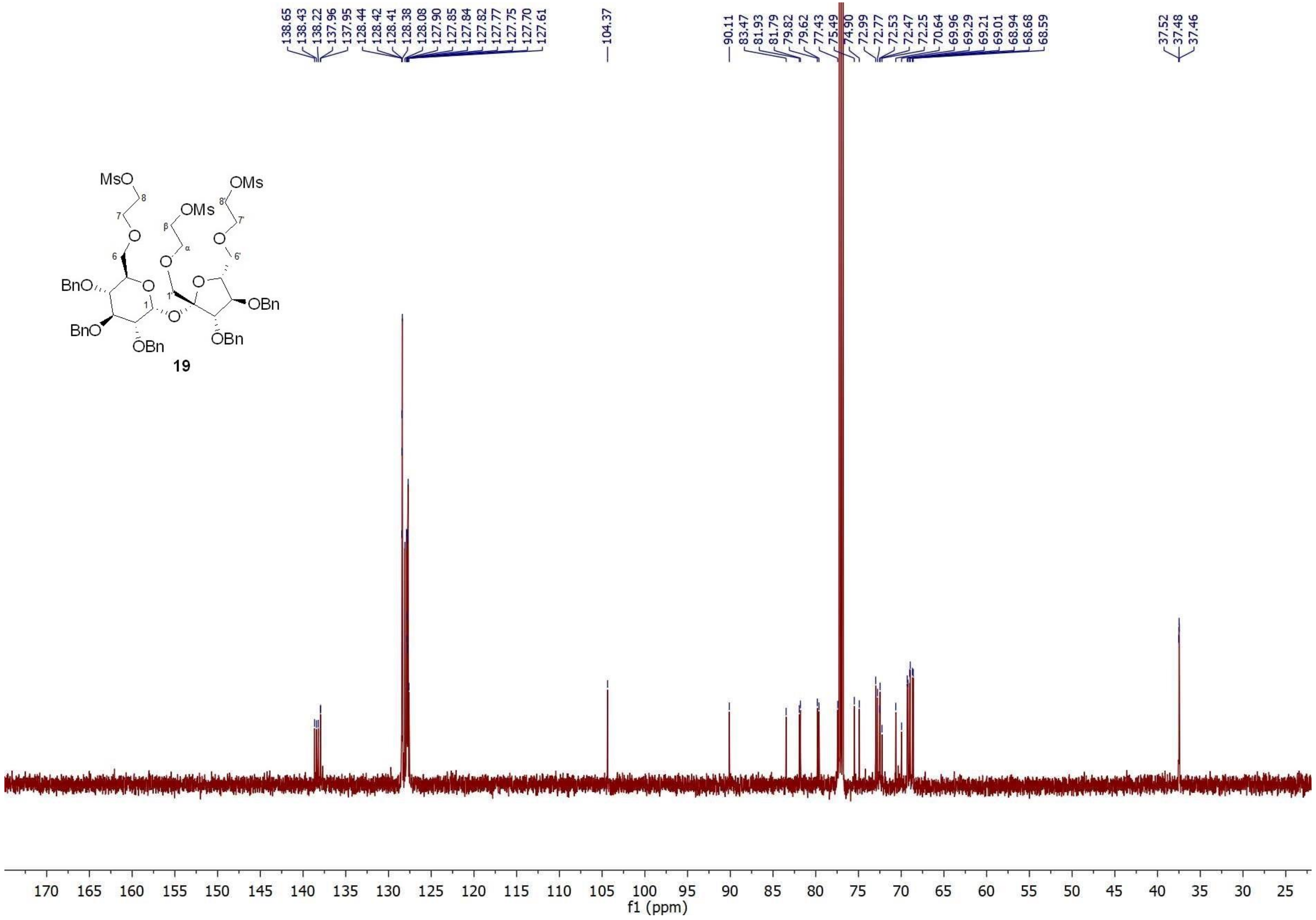

Figure S22 ^{13}C NMR spectrum of compound **19** ($CDCl_3$).

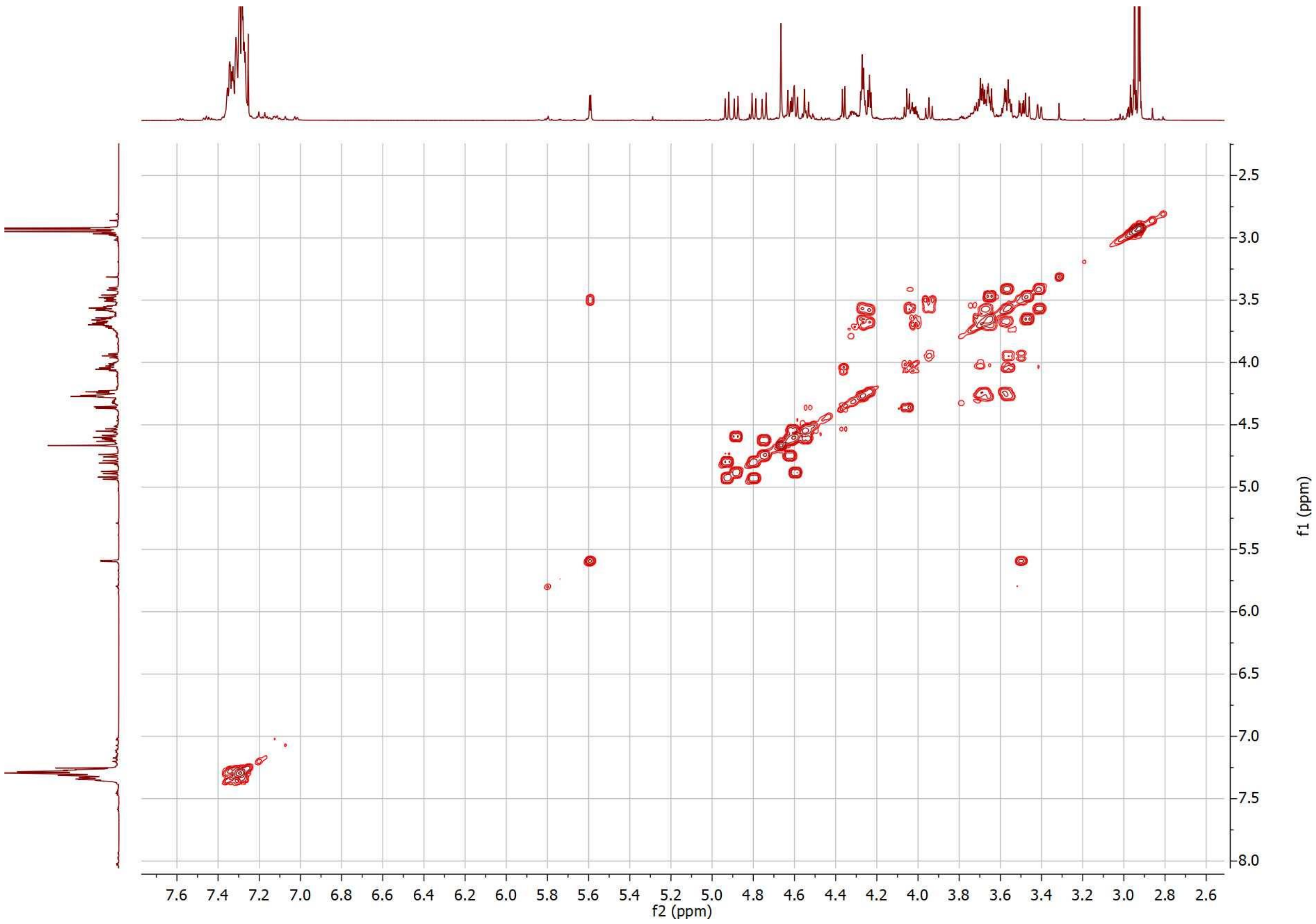

Figure S23 gCOSY spectrum of compound **19**.

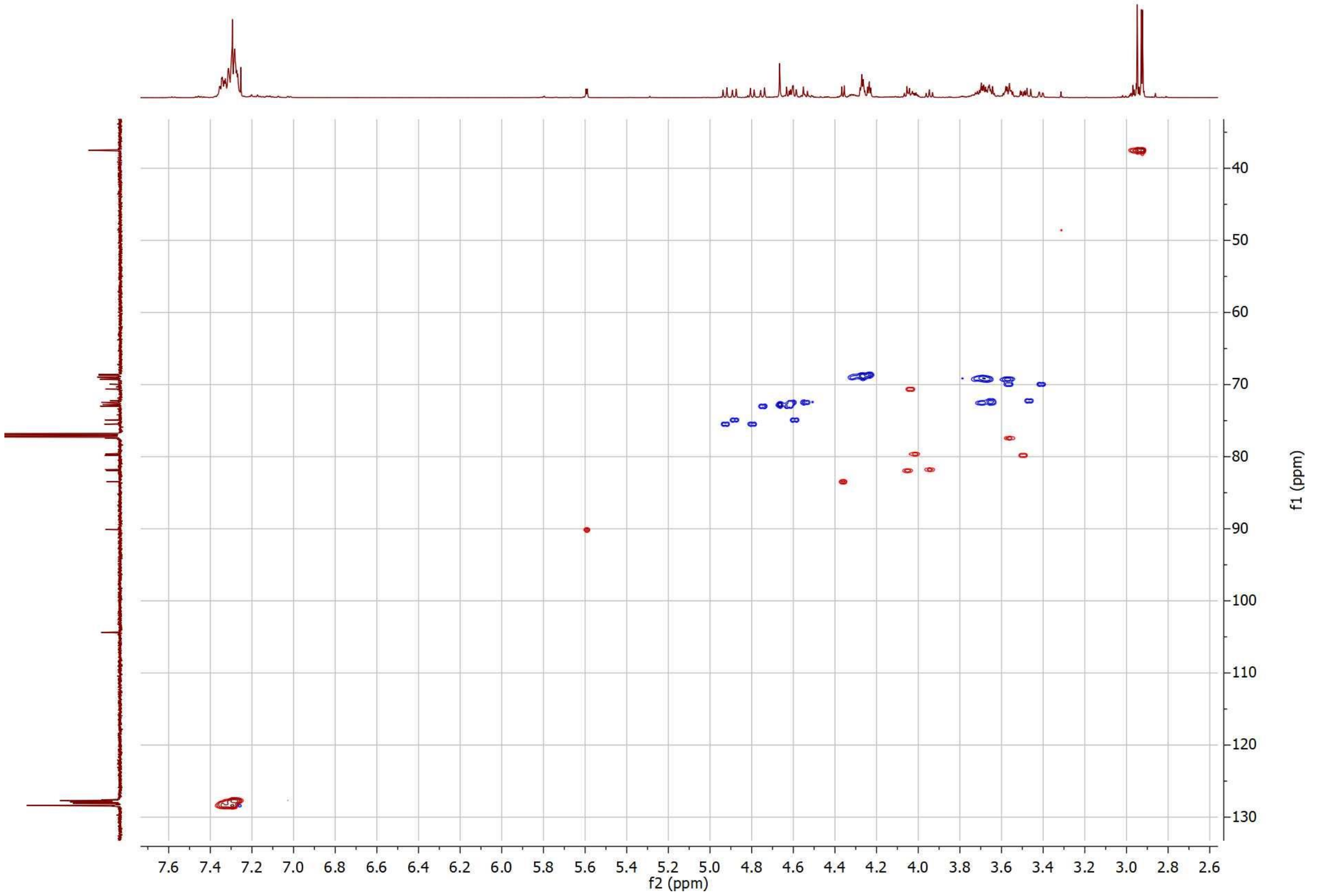

Figure S24 gHSQCAD spectrum of compound **19**.

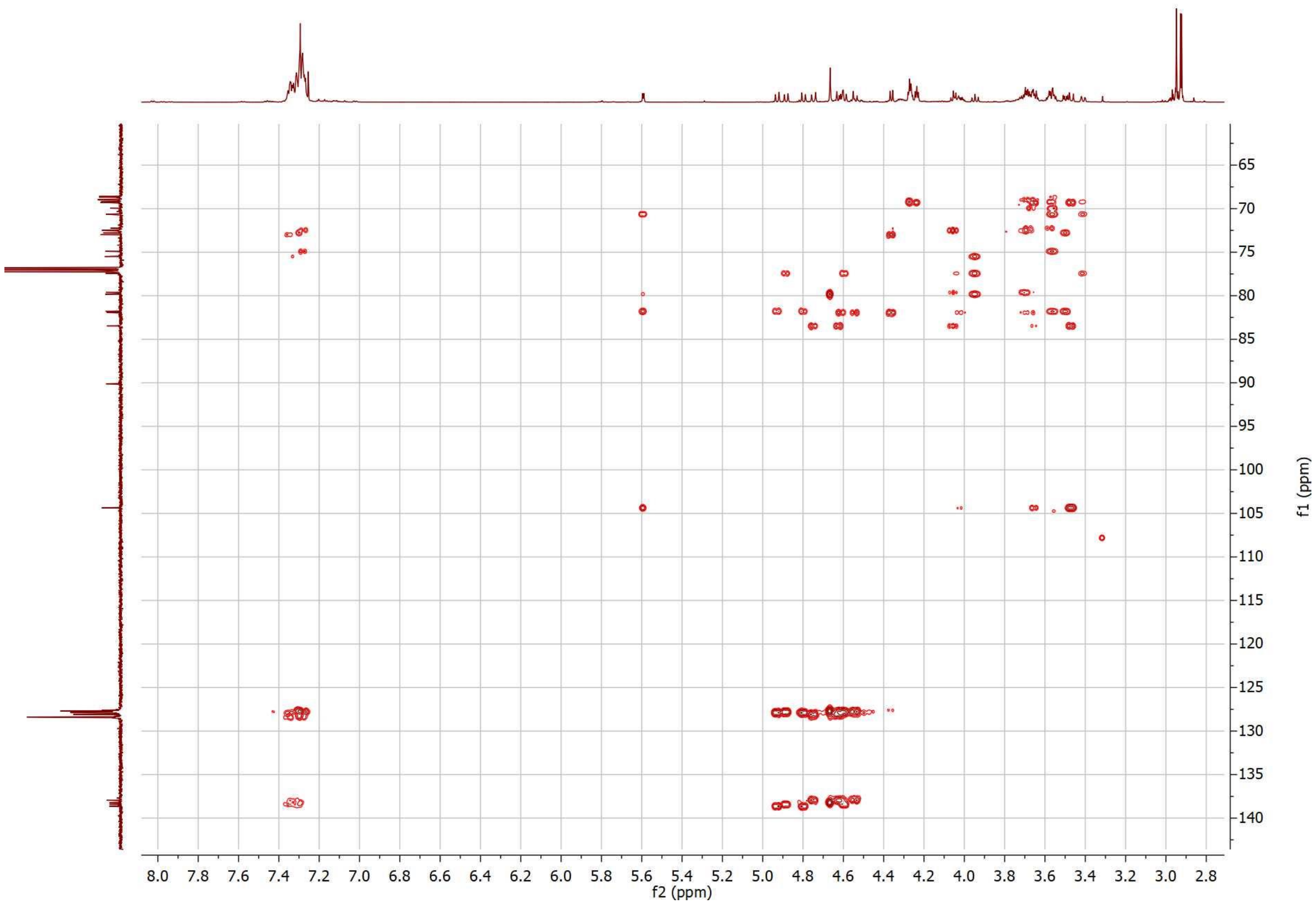

Figure S25 gHMBCAD spectrum of compound **19.**

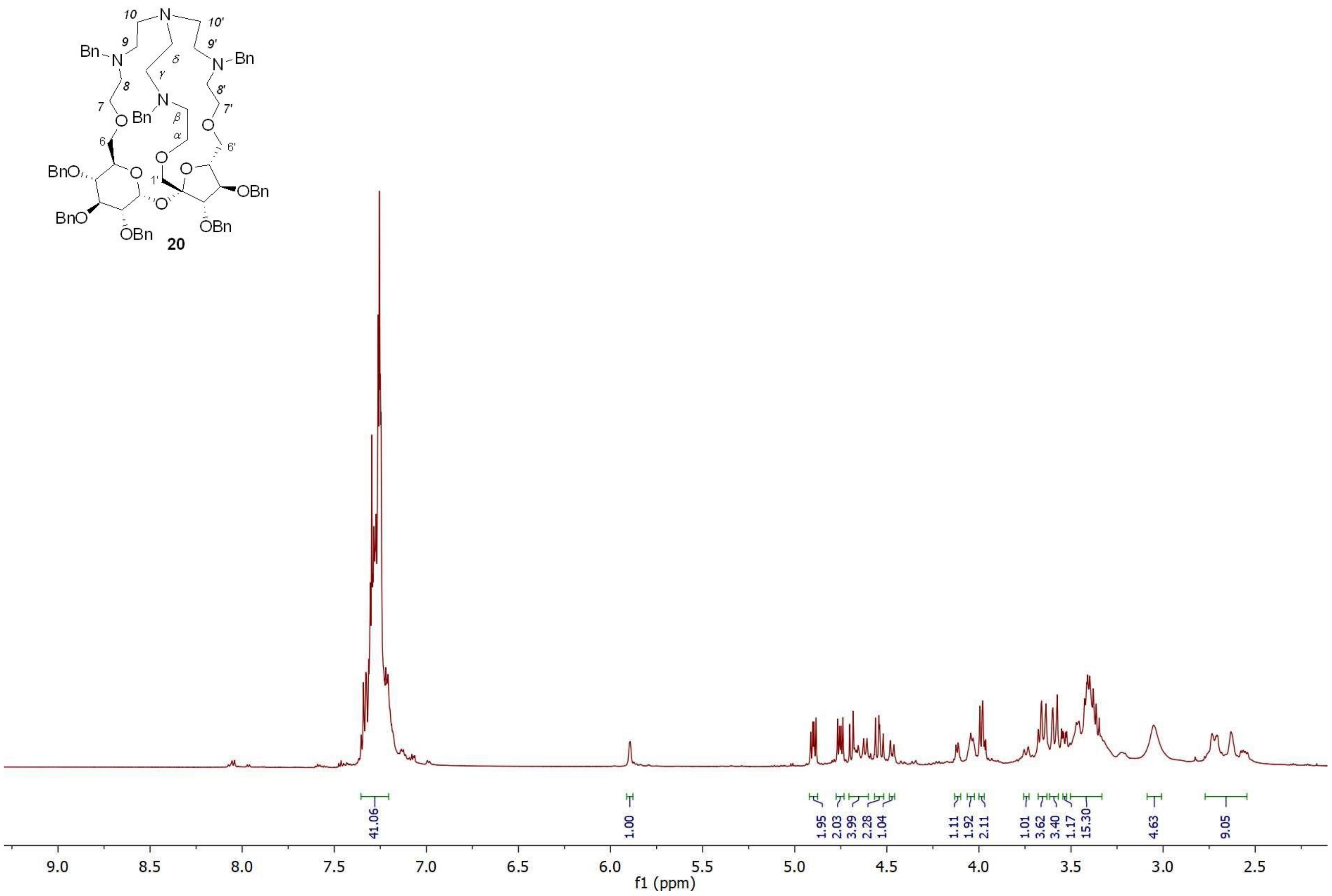

Figure S26 ^{1}H NMR spectrum of compound **20** ($CDCl_3$).

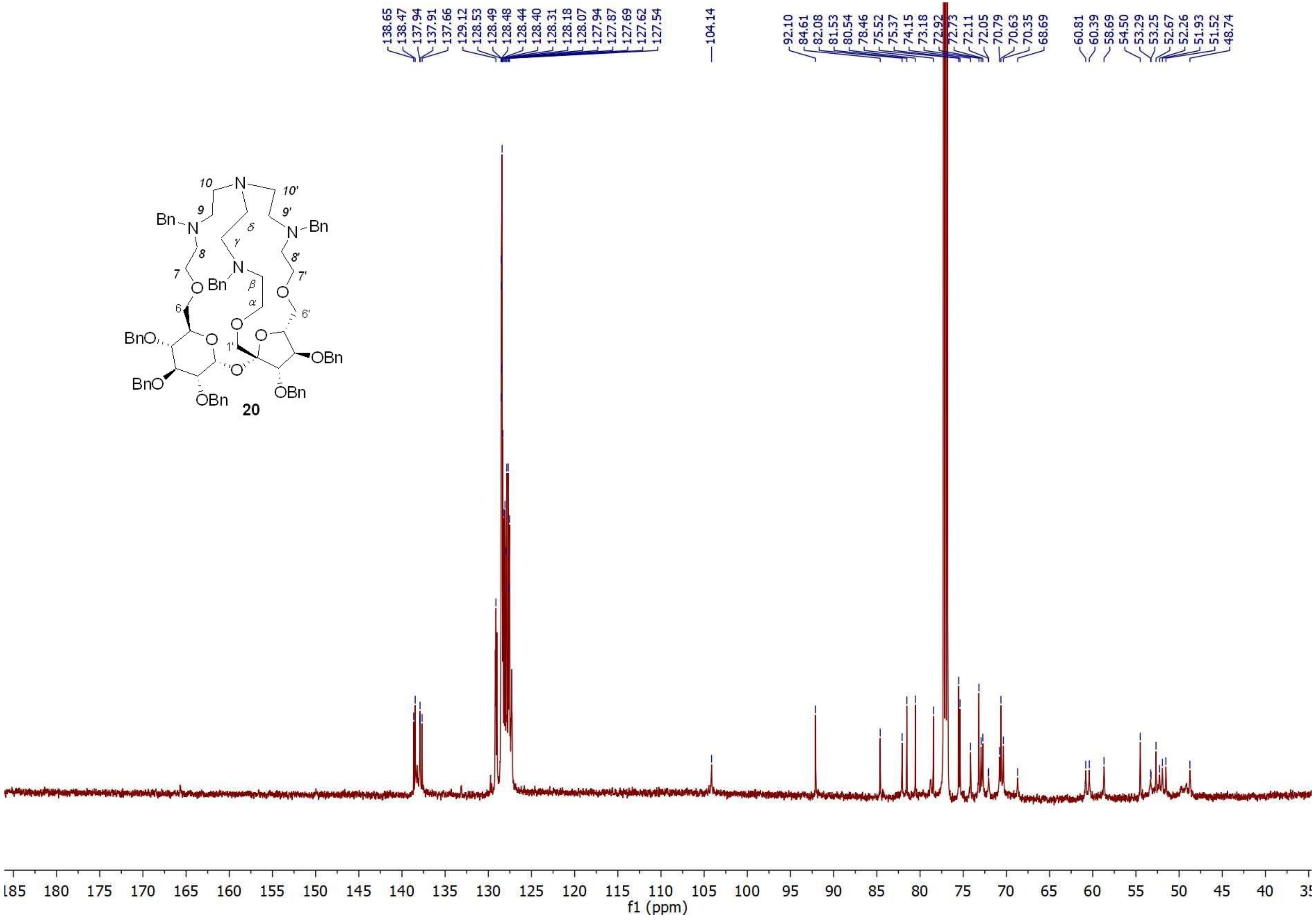

Figure S27 ^{13}C NMR spectrum of compound **20** ($CDCl_3$).

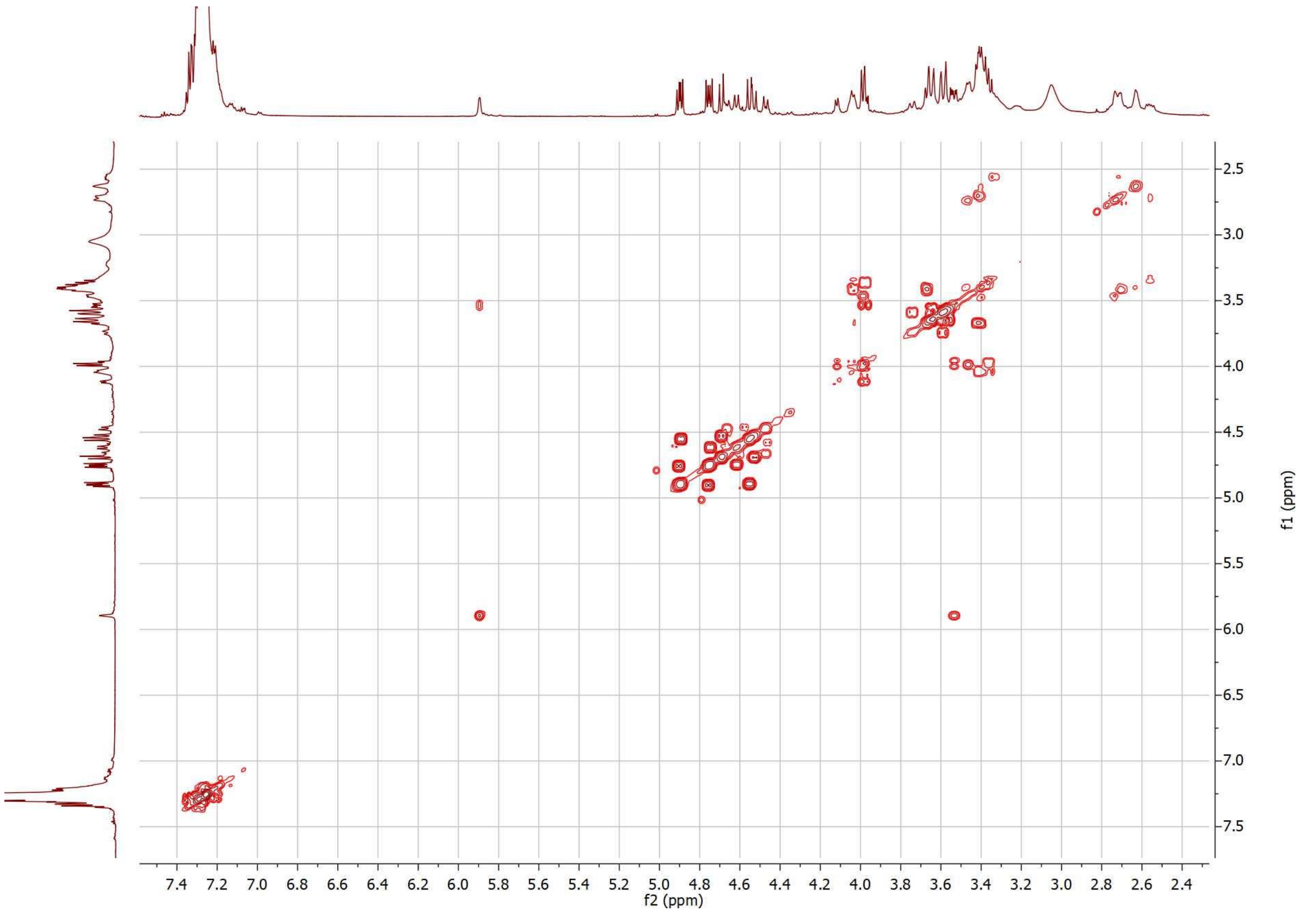

Figure S28 gCOSY spectrum of compound **20**.

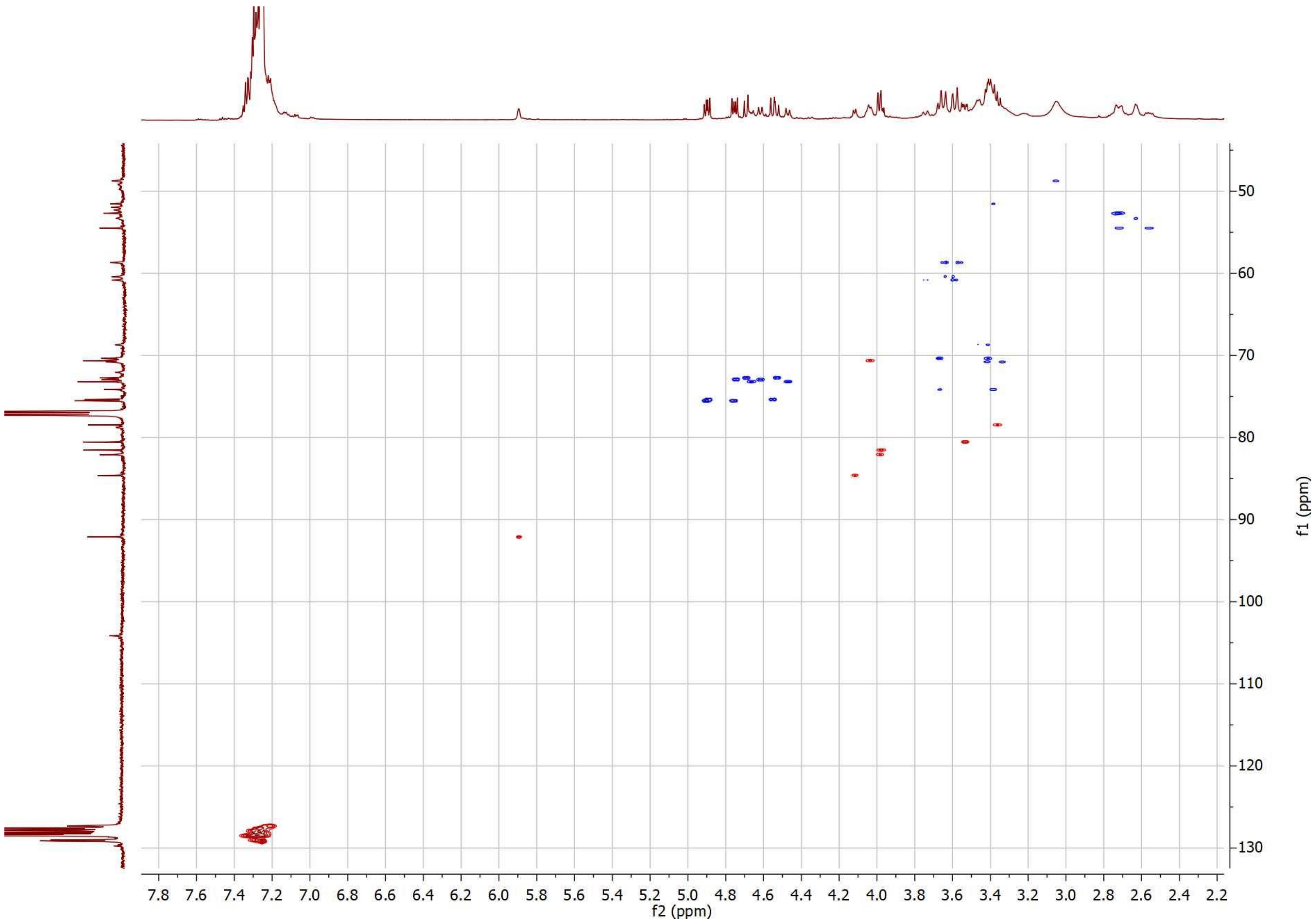

Figure S29 gHSQCAD spectrum of compound **20**.

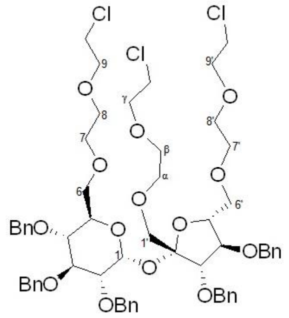

21

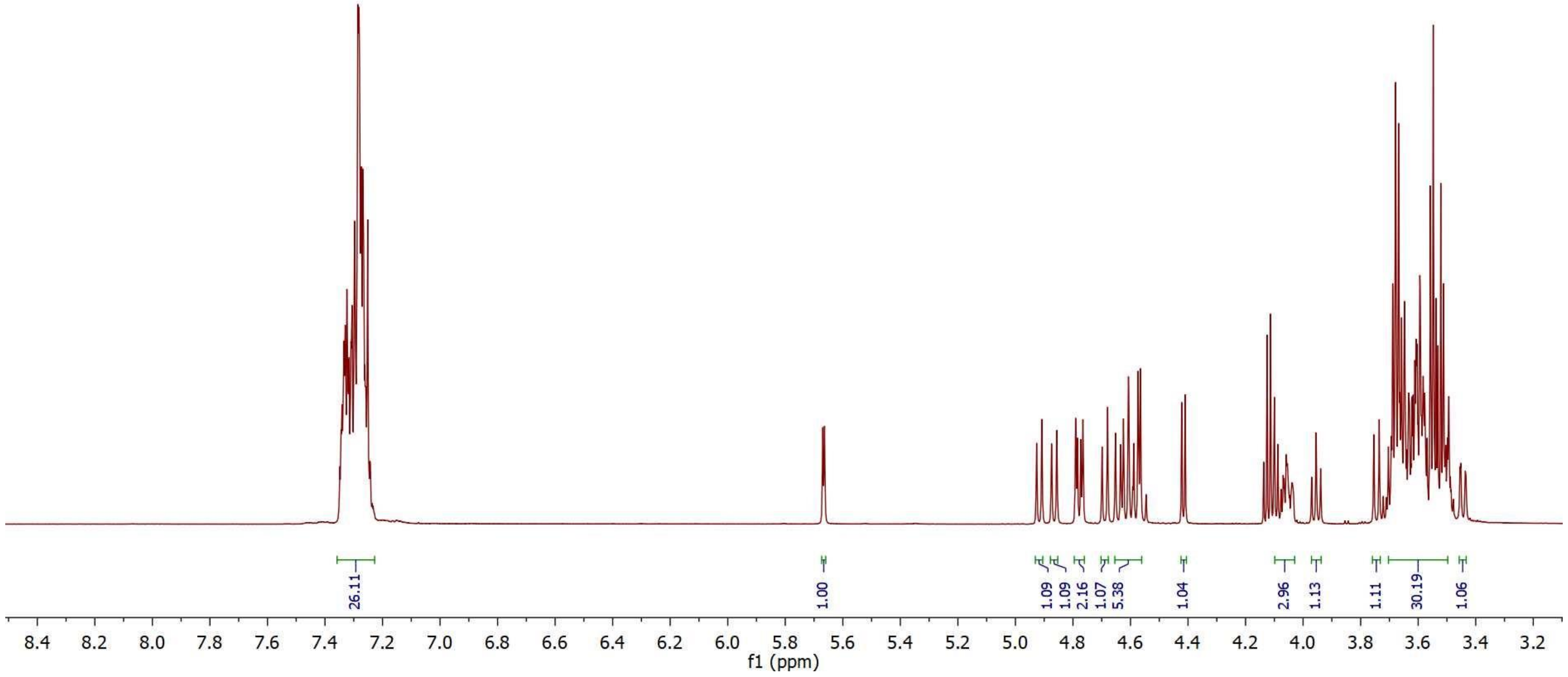

Figure S30 ^{1}H NMR spectrum of compound **21** ($CDCl_3$).

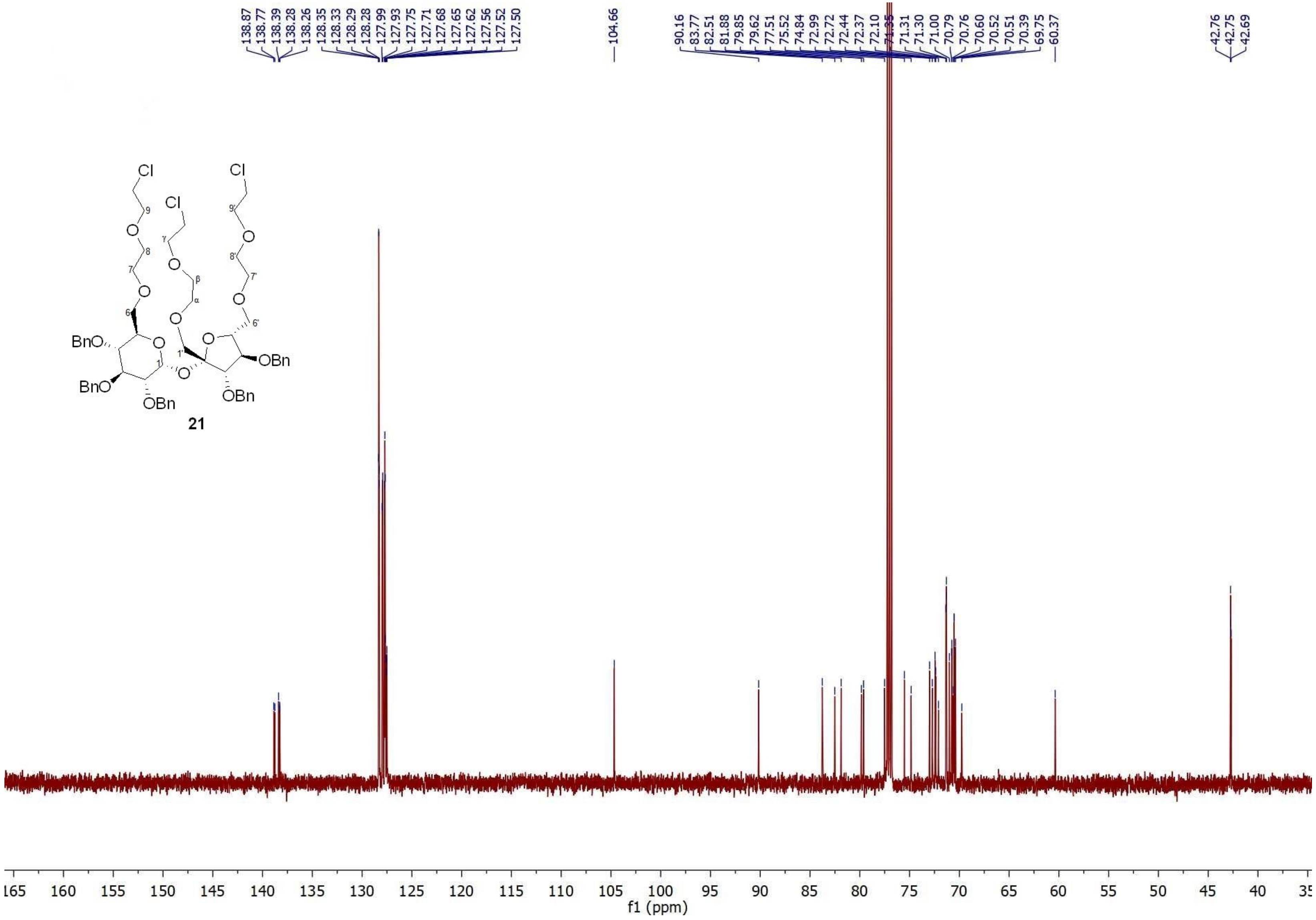

21

Figure S31 ^{13}C NMR spectrum of compound **21** ($CDCl_3$).

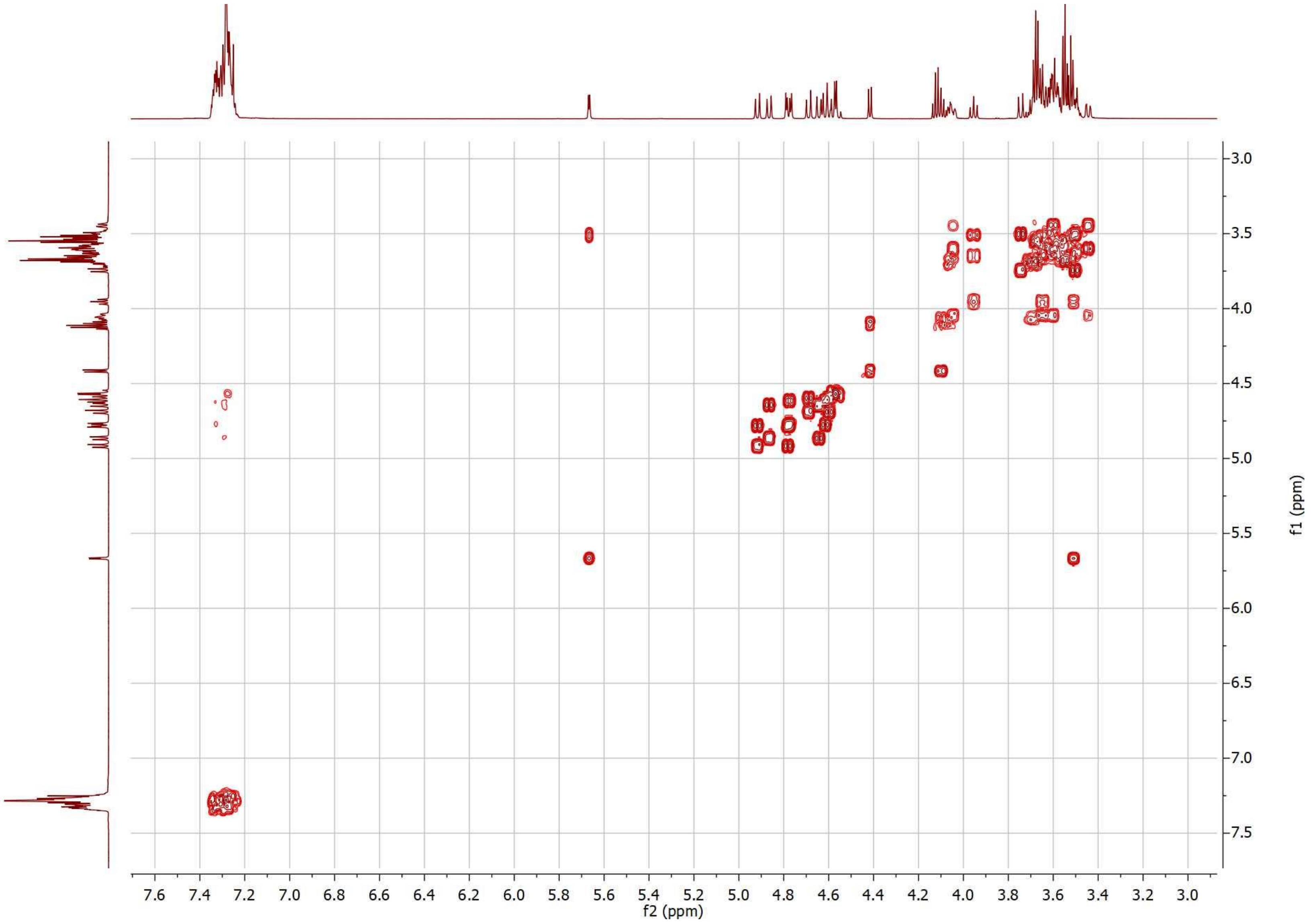

Figure S32 gCOSY spectrum of compound **21**.

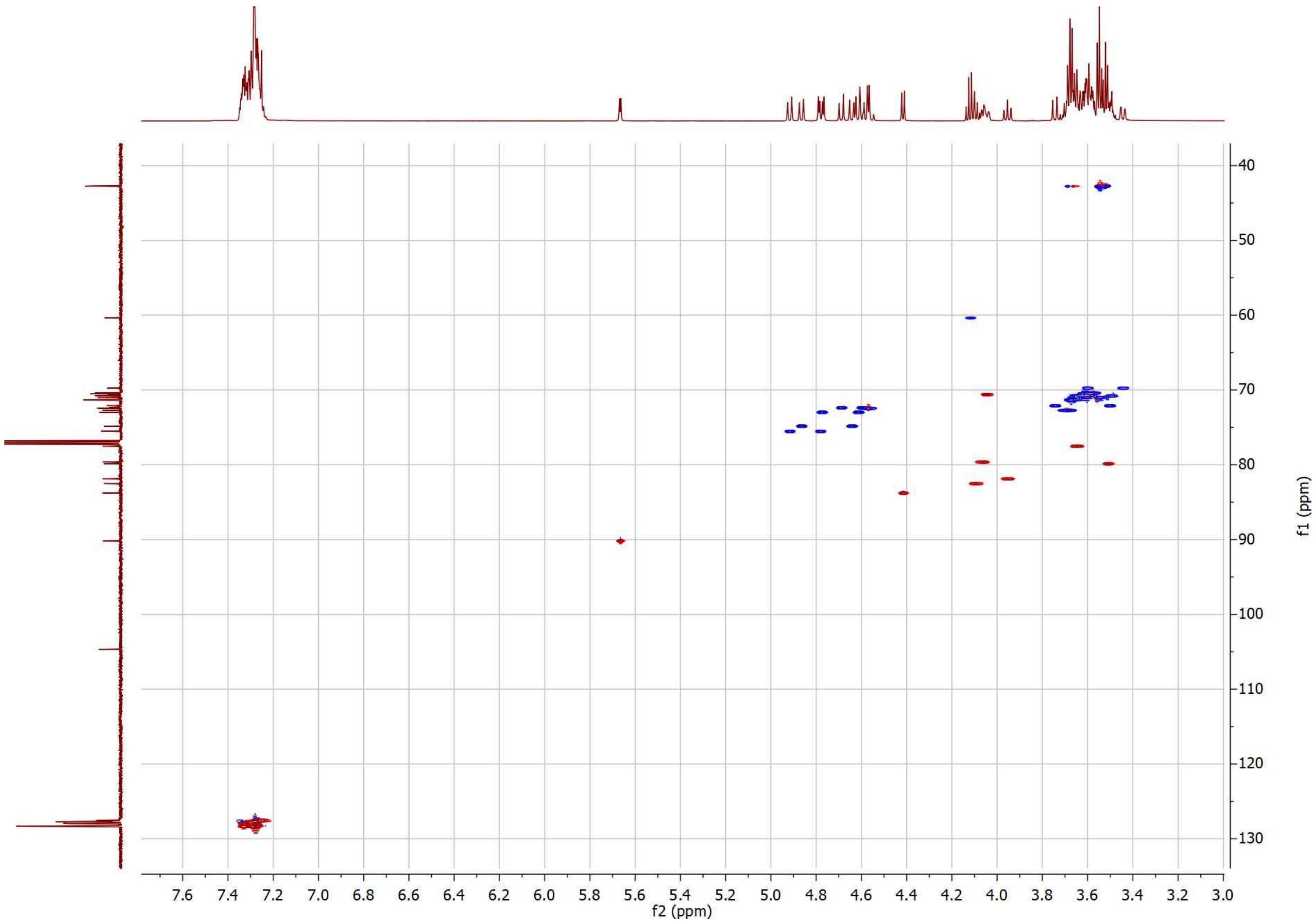

Figure S33 gHSQCAD spectrum of compound **21**.

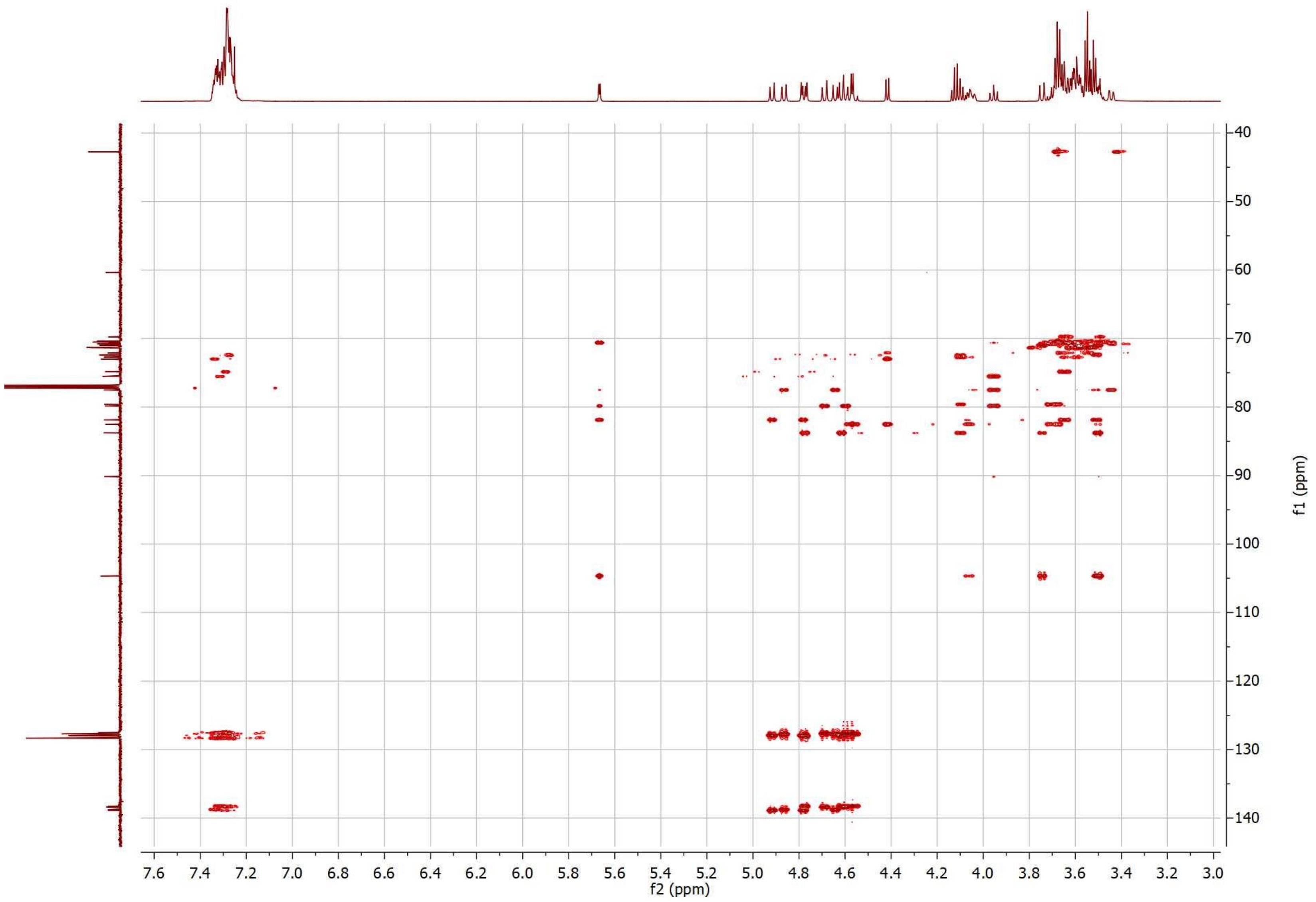

Figure S34 gHMBCAD spectrum of compound **21**.

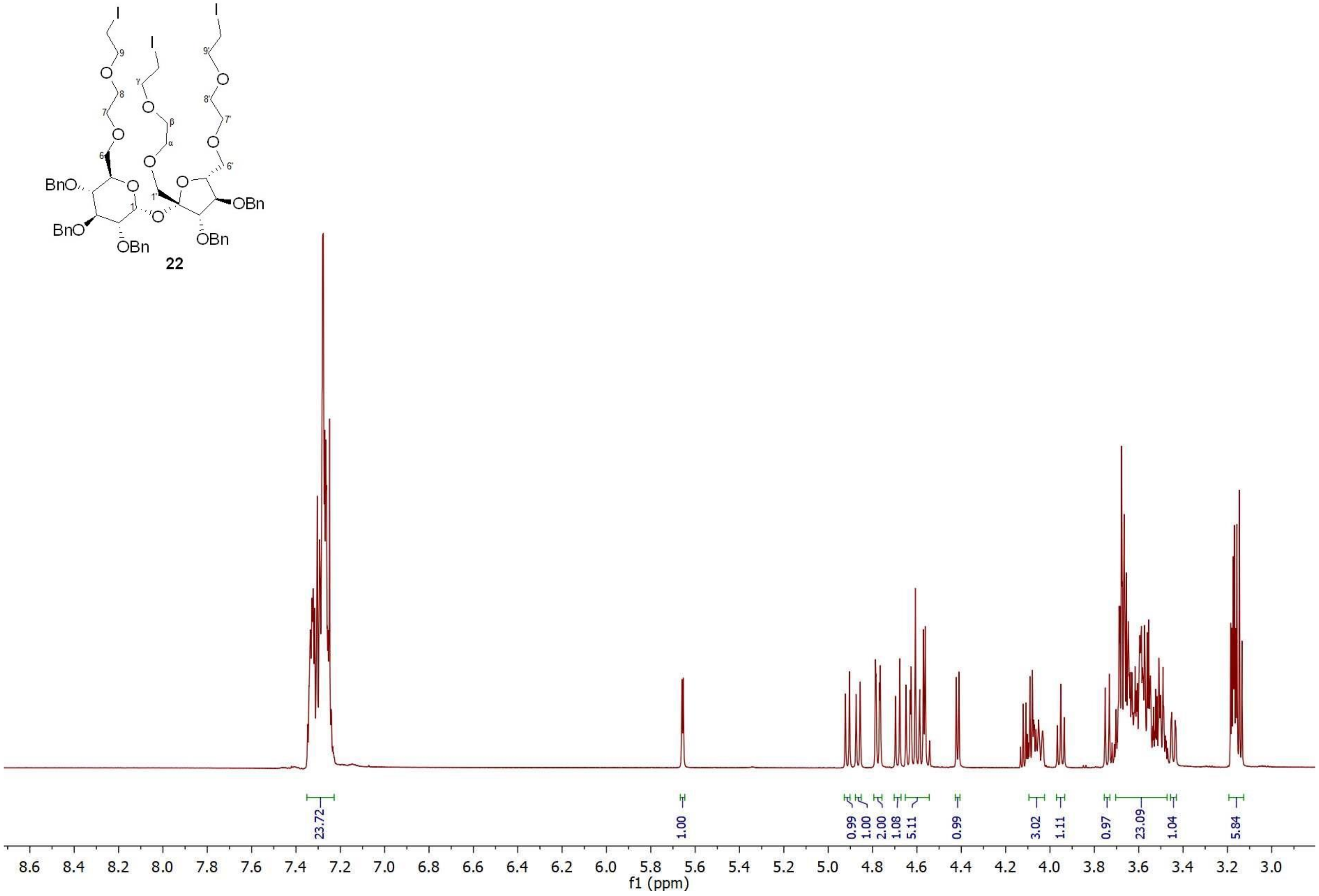

Figure S35 ^{1}H NMR spectrum of compound **22** ($CDCl_3$).

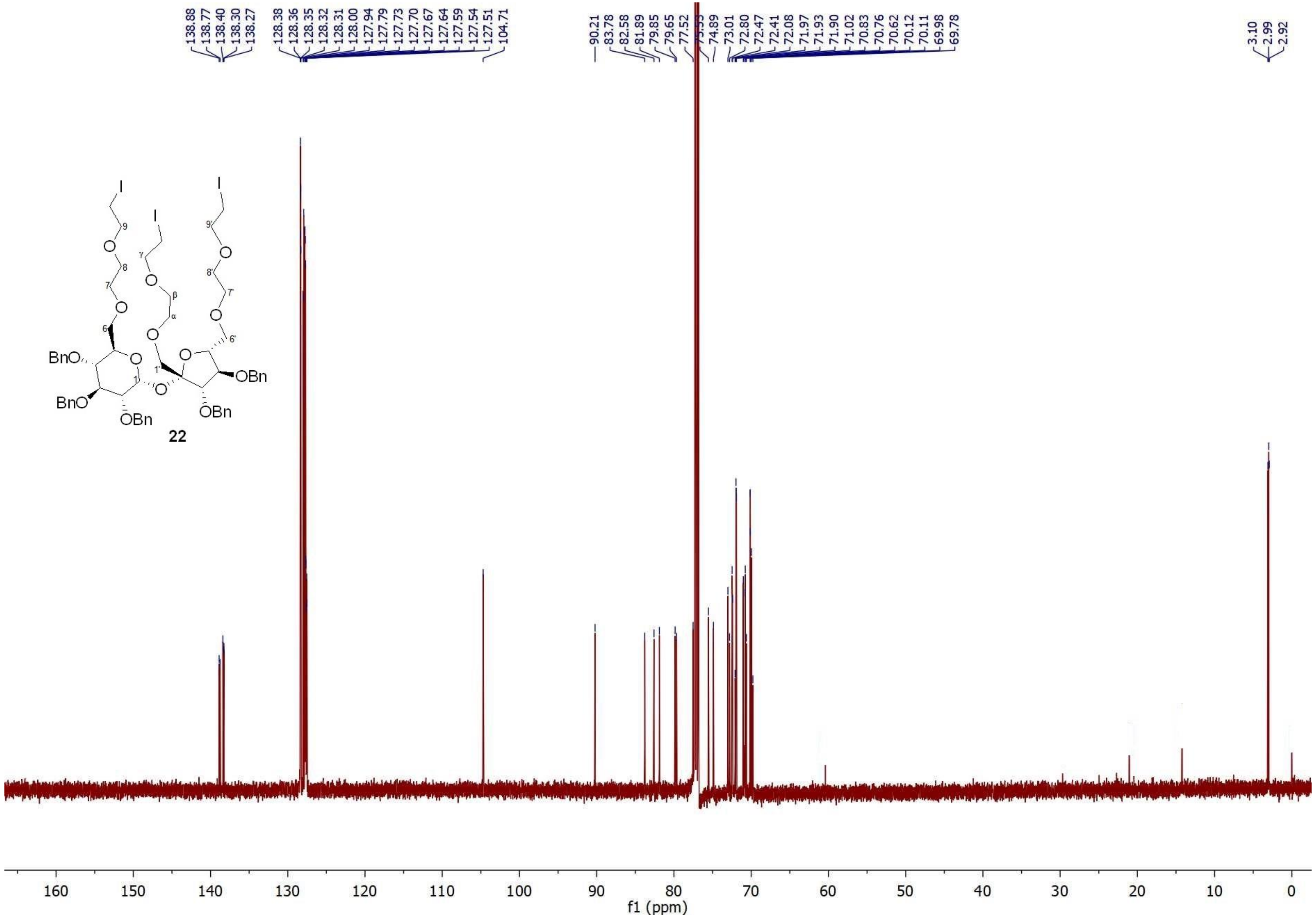

Figure S36 ^{13}C NMR spectrum of compound **22** ($CDCl_3$).

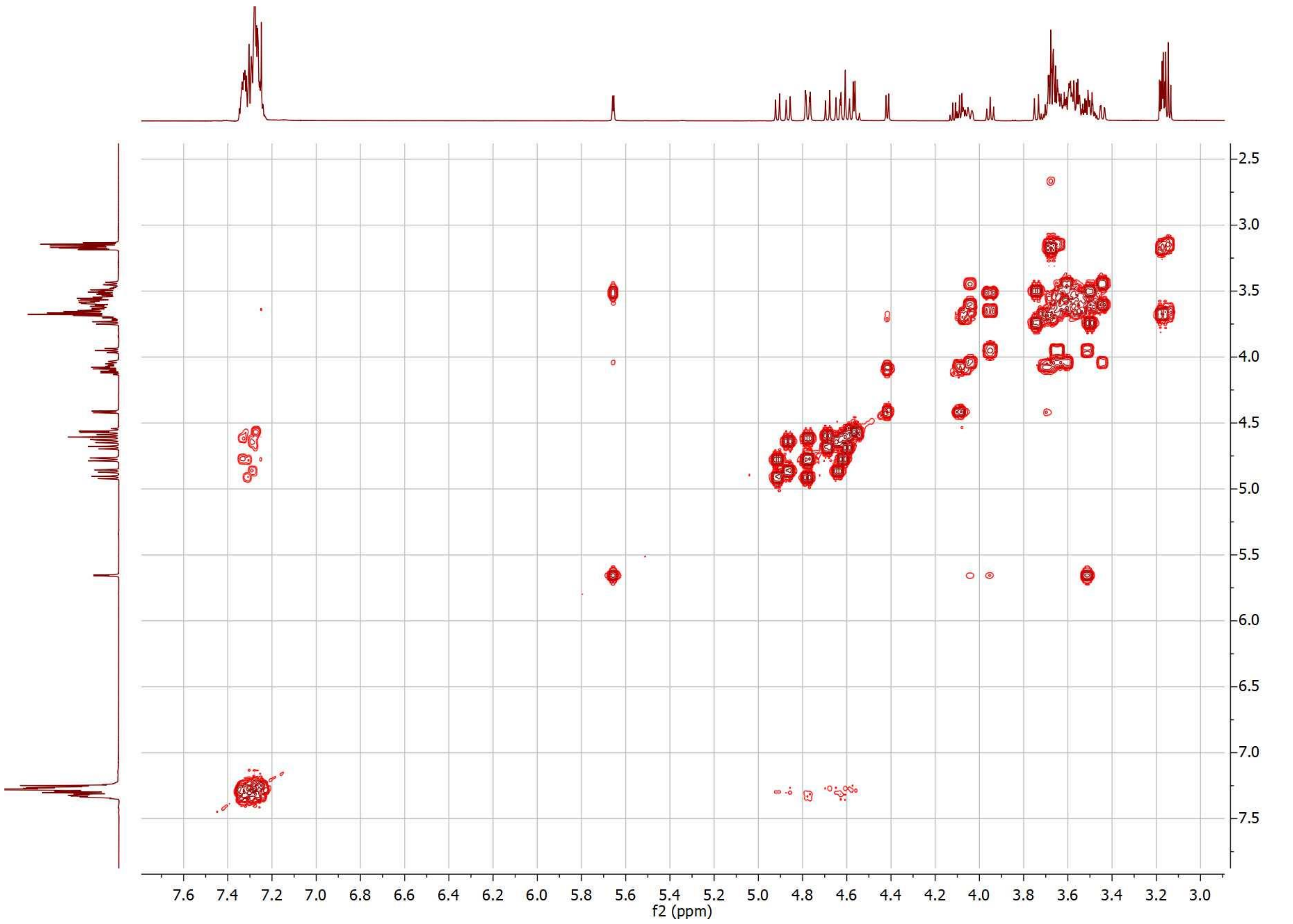

Figure S37 gCOSY spectrum of compound **22**.

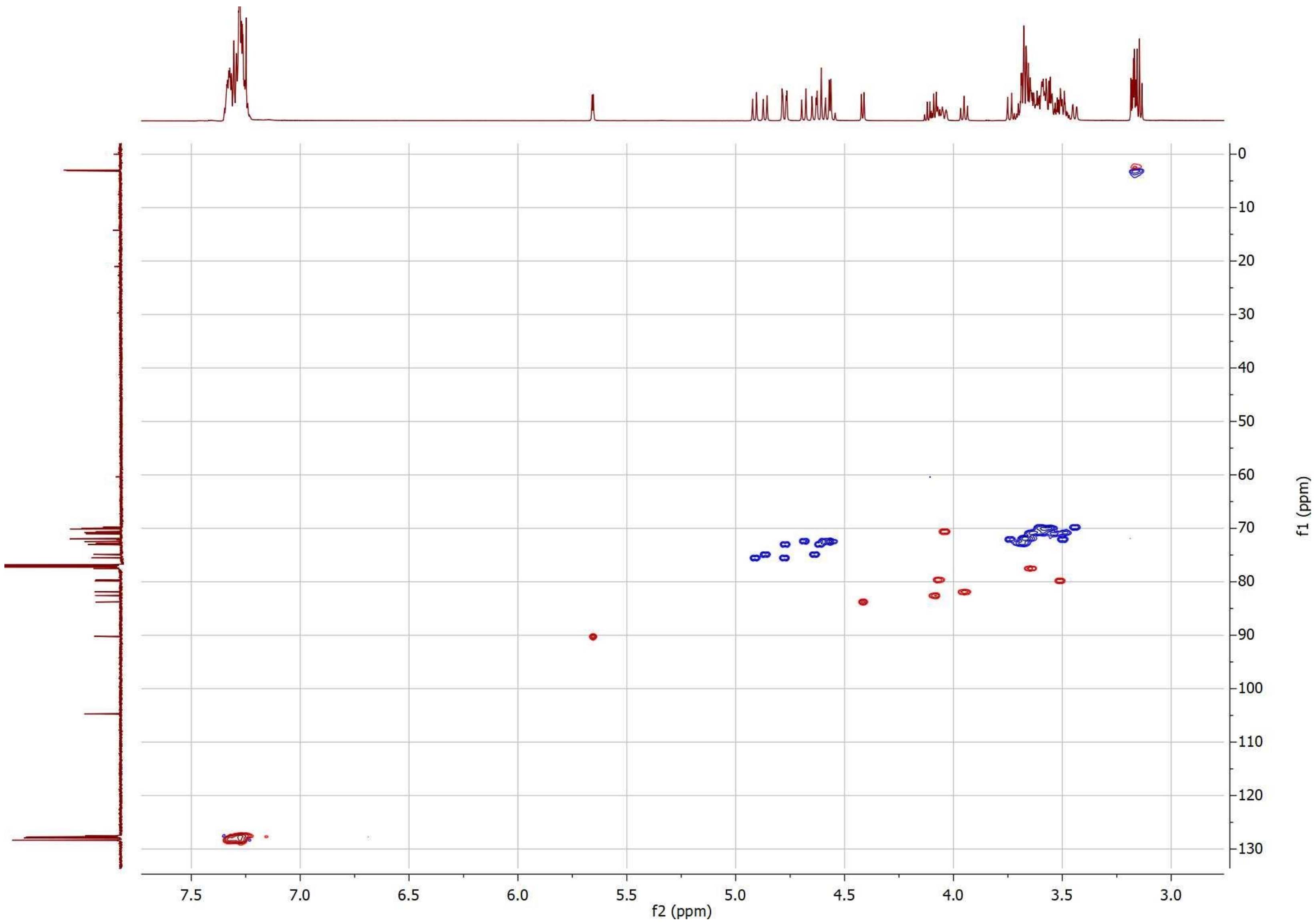

Figure S38 gHSQCAD spectrum of compound **22**.

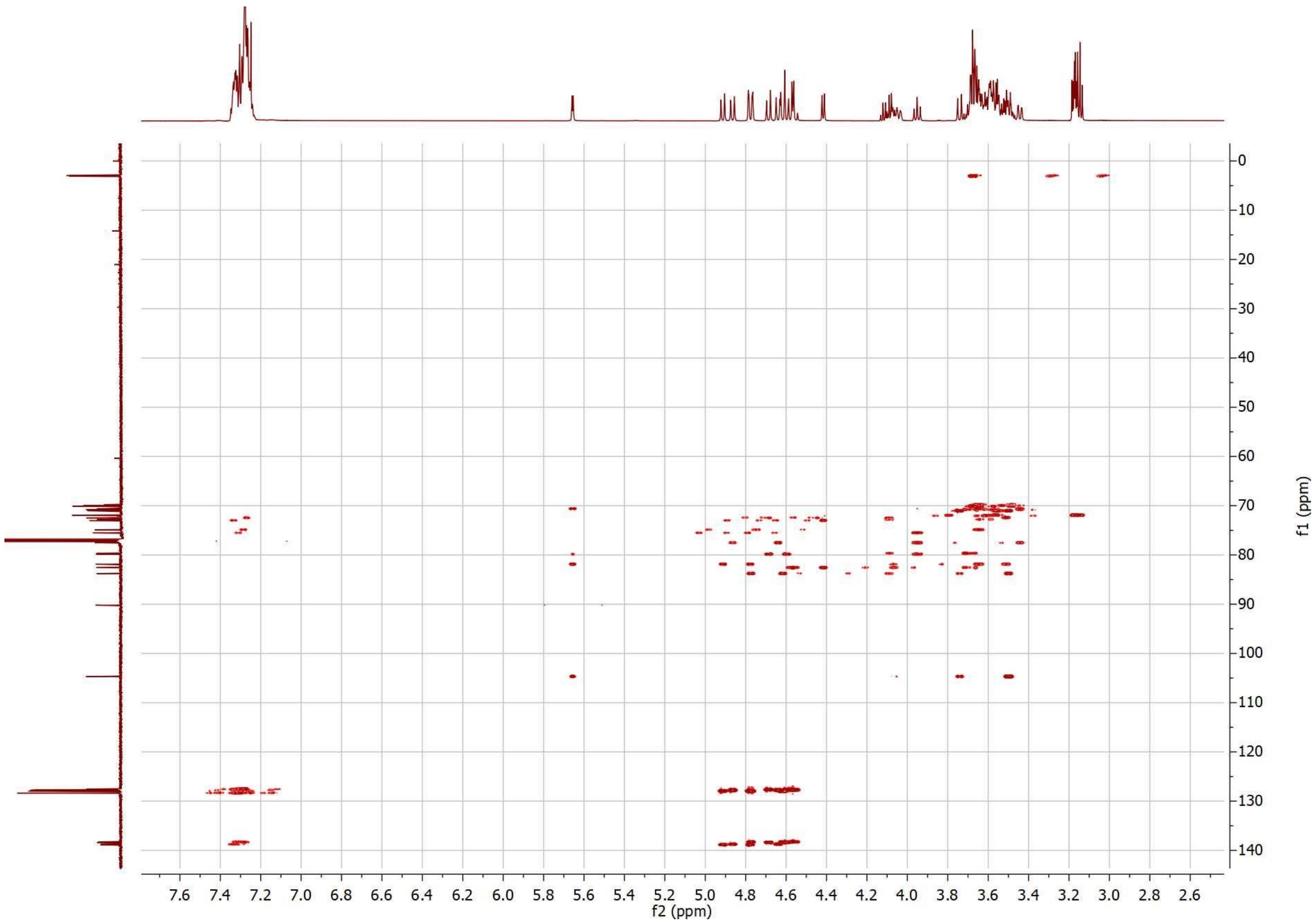

Figure S39 gHMBCAD spectrum of compound **22**.

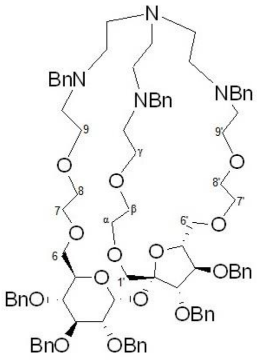

23

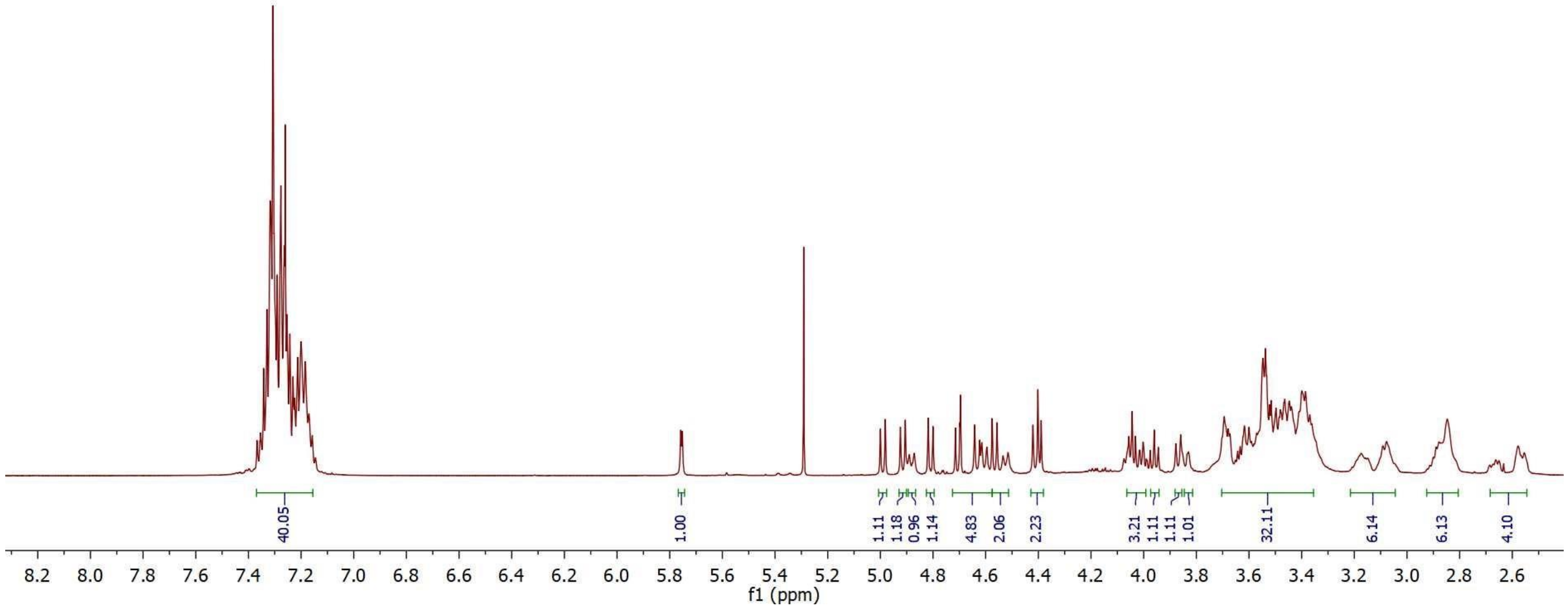

Figure S40 ^{1}H NMR spectrum of compound **23** (acetone-d_6).

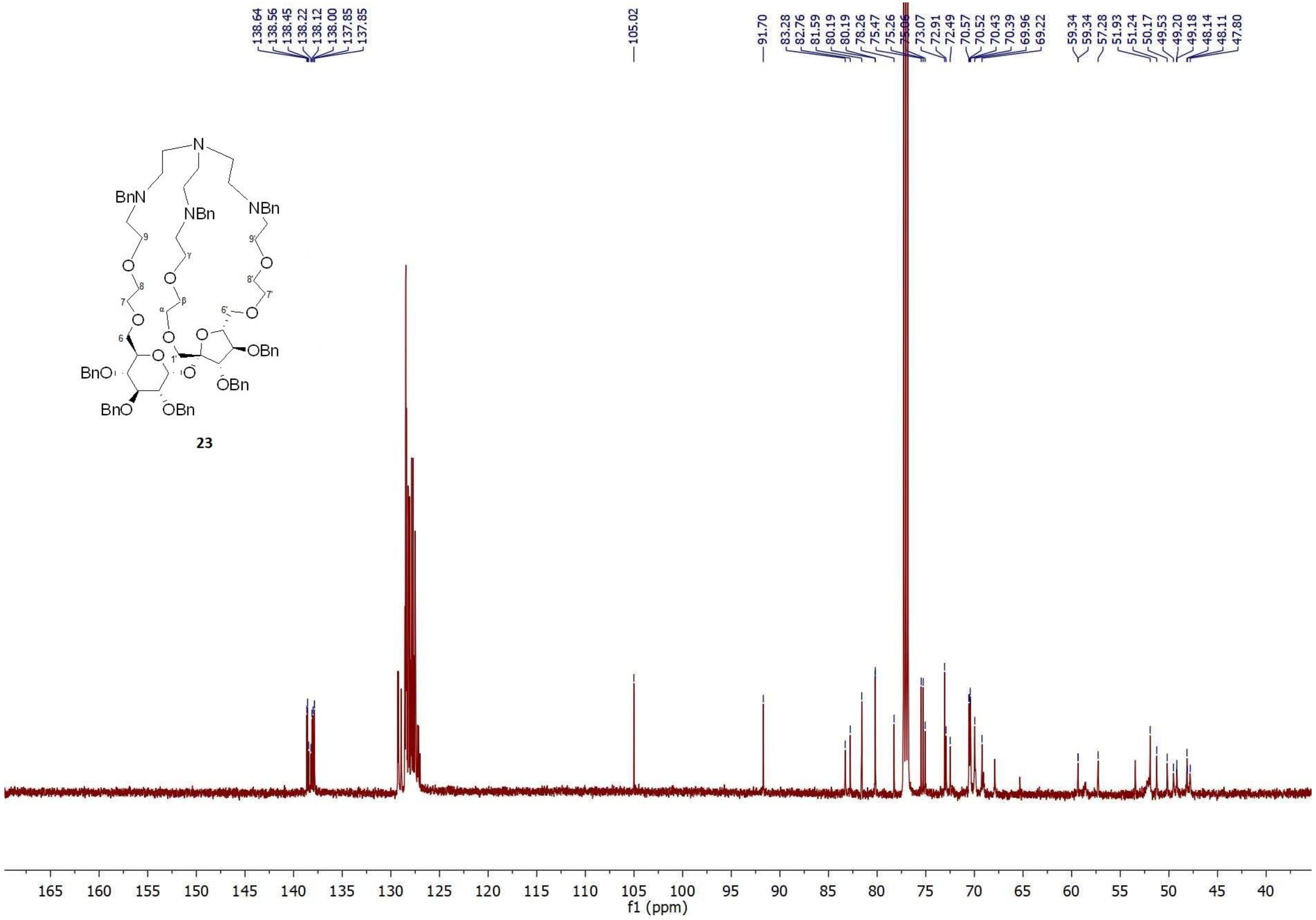

Figure S41 ^{13}C NMR spectrum of compound **23** (acetone-d_6).

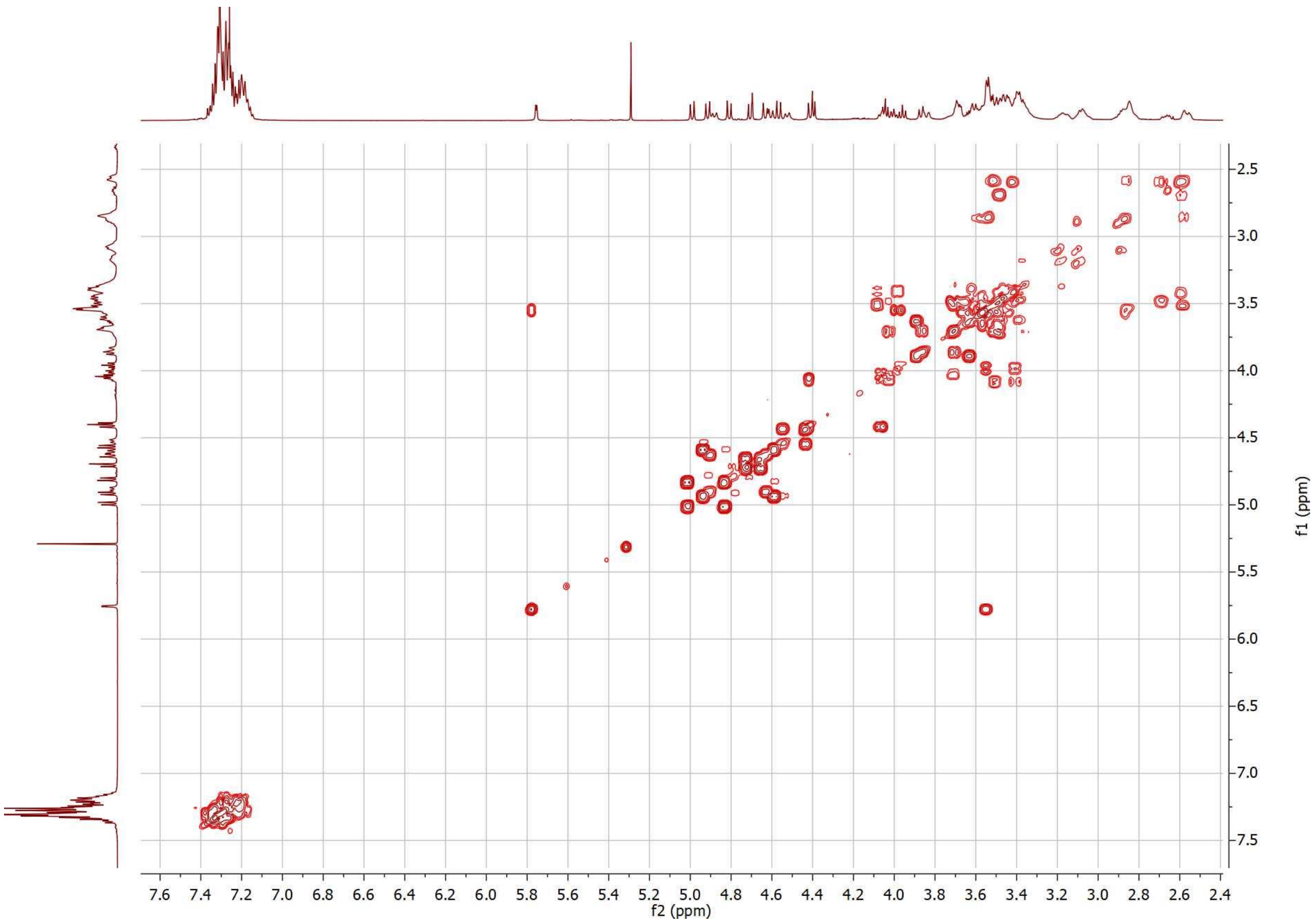

Figure S42 gCOSY spectrum of compound **23**.

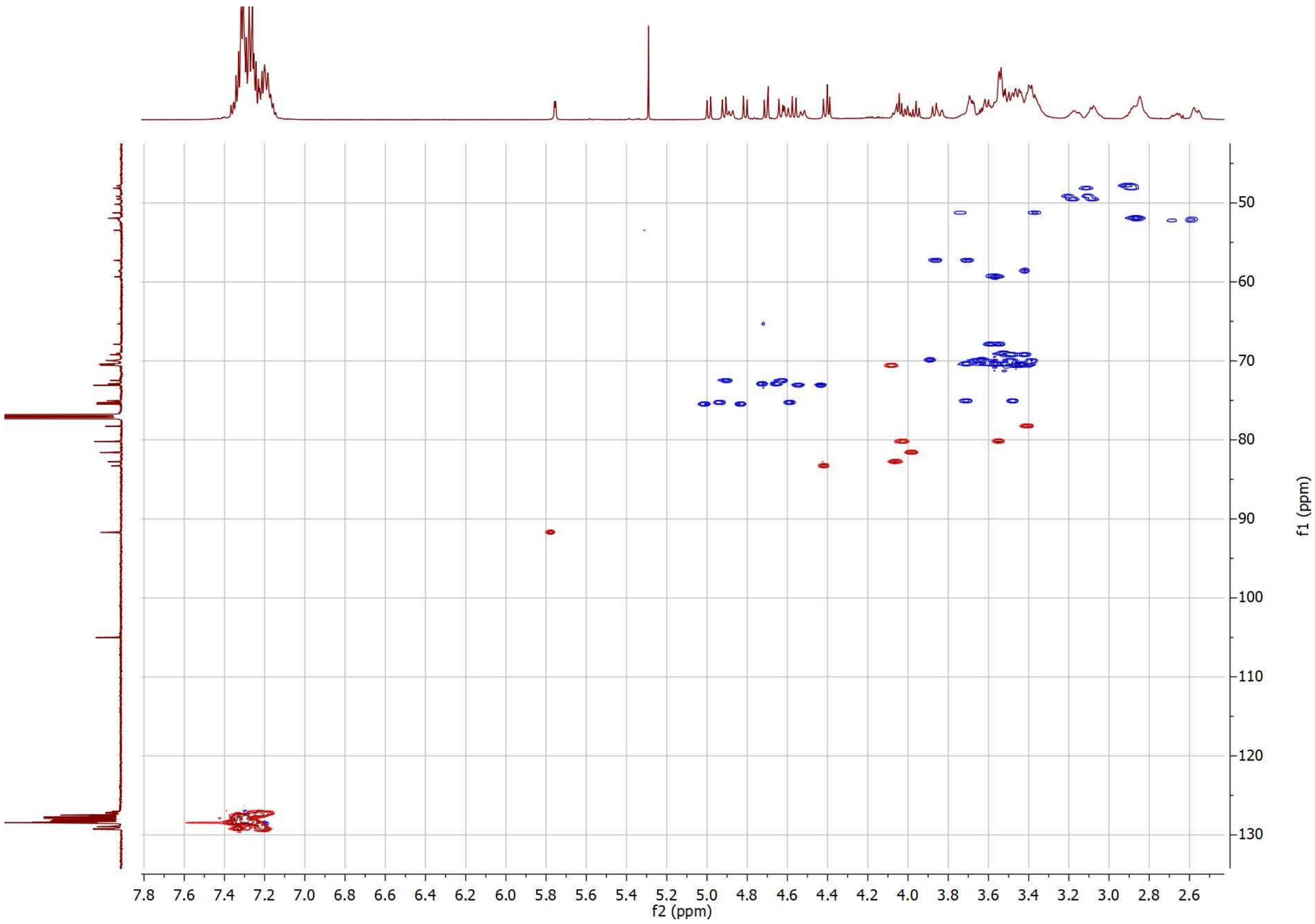

Figure S43 gHSQCAD spectrum of compound **23**.
